# Effects of Halogenation on Cyclopentadithiophenevinylene-Based Acceptors with Excellent Responses in Binary Organic Solar Cells

**DOI:** 10.1021/acsami.3c01487

**Published:** 2023-04-19

**Authors:** Fernando G. Guijarro, Pilar de la Cruz, Kanupriya Khandelwal, Rahul Singhal, Fernando Langa, Ganesh D. Sharma

**Affiliations:** † Instituto de Nanociencia, Universidad de Castilla-La Mancha, Nanotecnología y Materiales Moleculares (INAMOL), Campus de la Fábrica de Armas, 45071 Toledo, Spain; ‡ Department of Physics, The LNM Institute of Information Technology, Jamdoli, 302031 Jaipur, (Rai), India; § Department of Physics, Malviya National Institute of Technology, JLN Marg, 302017 Jaipur, (Raj.), India; ∥ Department of Electronics and Communication Engineering, The LNM Institute of Information Technology, Jamdoli, 302031 Jaipur, (Rai), India

**Keywords:** binary organic solar cells, cyclopentadithiophenevinylene, halogenated end-cap acceptors, PBDB-T donor polymer

## Abstract

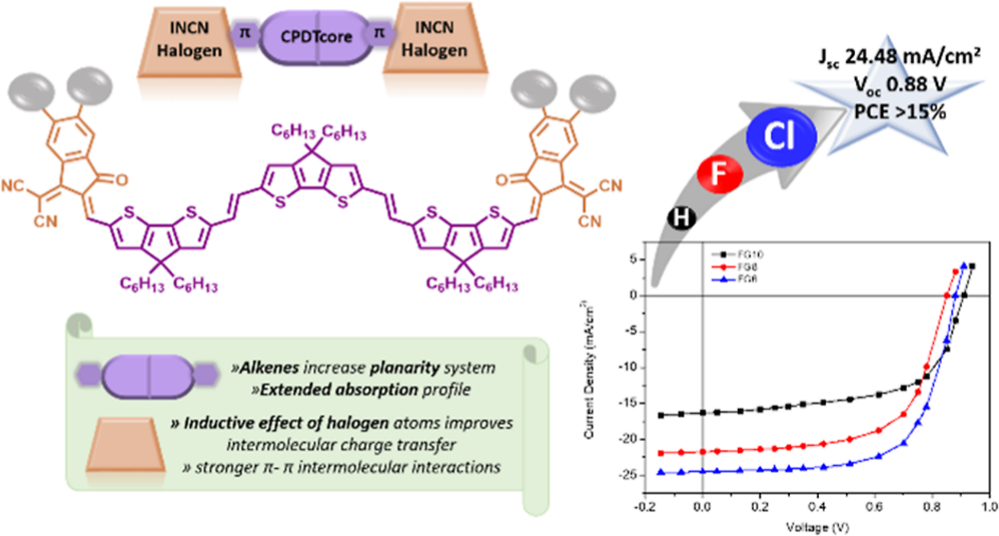

In recent years, non-fused non-fullerene acceptors (NFAs) have attracted increasing consideration due to several advantages, which include simple preparation, superior yield, and low cost. In the work reported here, we designed and synthesized three new NFAs with the same cyclopentadithiophenevinylene (CPDTV) trimer as the electron-donating unit and different terminal units (IC for **FG10**, IC-4F for **FG8**, and IC-4Cl for **FG6**). Both halogenated NFAs, i.e., **FG6** and **FG8**, show red-shifted absorption spectra and higher electron mobilities (more pronounced for **FG6**) in comparison with **FG10**. Moreover, the dielectric constants of these materials also increased upon halogenation of the IC terminal units, thus leading to a reduction in the exciton binding energy, which is favorable for dissociation of excitons and subsequent charge transfer despite the driving force (highest occupied molecular orbital and lowest unoccupied molecular orbital offsets) being very small. The organic solar cells (OSCs) constructed using these acceptors and PBDB-T, as the donor, showed PCE values of 15.08, 12.56, and 9.04% for **FG6**, **FG8**, and **FG10**, respectively. The energy loss for the **FG6**-based device was the lowest (0.45 eV) of all the devices, and this may be attributed to it having the highest dielectric constant, which leads to a reduction in the binding energy of exciton and a small driving force for hole transfer from **FG6** to PBDB-T. The results indicate that the NFA containing the CPDTV oligomer core and halogenated terminal units can efficiently spread the absorption spectrum to the NIR zone. Non-fused NFAs have a bright future in the quest to obtain efficient OSCs with low cost for marketable purposes.

## Introduction

Organic solar cells (OSCs) structured with bulk heterojunction (BHJ) developed increased consideration owing to their numerous benefits, which include semitransparency, low cost for large-area production, mechanical flexibility, and indoor applications.^1−8^ The BHJ thin film, used for the active layer, consists of a combination of donor and acceptor organic semiconducting materials that impart suitable phase separation for effective dissociation of excitons into charge carriers, followed by charge transport toward the electrodes. In the past, fullerene derivatives have been applied as acceptors due to their high electron mobility and suitable energy levels matched with donors, and OSCs prepared with fullerene acceptors have attained power conversion efficiencies (PCEs) of around 11%.^9^ Nevertheless, there are inherent disadvantages to fullerene-based devices, and these include the weak and narrow absorption range, difficulty in tuning the highest occupied molecular orbital (HOMO), and the high energy loss. However, in the last few years, numerous advances have been made in the design of OSCs, and this is attributed to the development of new light-harvesting constituents. Among them, acceptor–donor–acceptor (A–D–A) non-fullerene small molecule acceptors (NFSMAs) have attracted significant attention owing to their unique properties of excellent charge separation and transport qualities, which originate from their exceptional frontier electron density distributions.^10−13^ In 2019, Zou et al. developed fused-ring A–DA′D–A Y-series acceptors, e.g., Y6, that represent a novel concept of NFSMAs,^14^ and subsequent chemical modification of the central DA’D units,^14−16^ side chains,^15−20^ and terminal acceptor units^21−23^ boosted the PCEs of OSCs toward 20%.^24−29^ However, the complicated synthesis, which gave a low global yield, particularly for the ring-closure reactions, and the high cost of fused ring acceptors are the most significant drawbacks for the large-scale use of these materials. NFSMAs based on non-fused rings have been considered as potential replacements for the fused systems in recent years.^30−37^ The simplified synthetic route could well equilibrium the trade-off between the price, effectiveness, and stability of NFSMAs.^38,39^ As a consequence, there is a need to develop non-fused ring NFSMAs that incorporate other structural blocks.

At present, most of the highly efficient NFSMAs contain large π-conjugated core groups, such as indoledithiophene, indacenodithieno[3,2-*b*]thiophene, or their derivatives. The synthesis of these cores requires multi-step processes that are not compatible with commercial applications. For OSCs, there is therefore a need to design new non-fused NFSMAs with simple and efficient synthetic routes. 4*H*-Cyclopenta[1,2-*b*:5,4-*b*′]dithiophene (CPDT) is an exceptional donor unit with a high HOMO energy level,^40^ and this unit has been used in small-molecule and polymer donor materials.^41−43^ The absorption profile can be expanded toward the longer wavelength region as a consequence of the rigid molecular structure of CPDT. Moreover, the electron richness of CPDT makes it feasible to prepare low-band gap polymers with electron acceptor groups through the push–pull effect.^42,44^ Additionally, the planar and extensive π-electron delocalization, in conjunction with the appropriate molecular stacking of this unit, ensures high charge transport attributes, and the branched alkyl chains impart good solubility in most organic solvents. The advantages of the CPDT unit outlined above mean that this structure has been used as a central electron-donating core in NFSMAs^45−47^ with resulting BHJ-PSC efficiencies in the range 9–10% using PBDB-T as the donor.^46,48^

In the study reported here, we designed and synthesized three new non-fused NFSMAs based on the same three-unit oligomer of CPDT as the core, in which the CPDT units are connected by double bonds, and different terminal units, namely, INCN-4Cl, INCN-4F, and INCN denoted as **FG6**, **FG8**, and **FG10**, respectively. The presence of the double bonds between the CPDT units increases the conjugation length, and this is beneficial in extending the absorption profile toward longer wavelength regions. The inclusion of double bonds also increases the planarity of the system. The influence of different halogens (Cl and F) attached to the INCN terminal units on the optical and electrochemical parameters was examined, and it was established that the optical band gap is reduced by the inclusion of the halogen. This reduction in band gap was more marked for Cl. The novel NFSMAs were used as acceptors along with PBDB-T as a donor to obtain information about the photovoltaic responses. The HOMO levels of these acceptors are in the range −5.25 to −5.30 eV, and the lowest unoccupied molecular orbital (LUMO) levels are in the range −3.89 to −3.96 eV. Therefore, PBDB-T was selected as the donor since its HOMO/LUMO (−5.21/–3.53 eV) levels matched those of the non-fullerene acceptors (NFAs) for efficient exciton dissociation and charge transfer. PBDB-T has been used as the donor along with fused^49−51^ and non-fused NFSMA^52−54^ and achieved the highest PCE of 15.85^55^ and 14.69%,^55^ respectively. Moreover, the absorption profile of PBDB-T and NFSMAs is complementary. After optimization of the systems, PCE values of 15.08, 12.57, and 9.04%, respectively, were obtained for PSCs based on **FG6**, **FG8**, and **FG10**, respectively. The highest value was obtained with **FG6**, and this may well be ascribed to the superior values of both, *J*_SC_ and FF, for this compound. Although the HOMO energy levels of **FG6** and **FG10** are similar, the higher value of *V*_OC_ for the **FG6-**based device when compared to **FG8** may be imputed to the larger dielectric constant, which in turn leads to lower exciton binding energy and the need for a lower driving force for hole transfer (HT) from the acceptor **FG6** to PBDB-T, thus resulting in a low energy loss.

## Results and Discussion

Compounds **FG6**, **FG8**, and **FG10** were obtained by the pyridine-catalyzed Knoevenagel reaction between bisaldehyde **1**(40) and 3-indanone derivatives **2a**,^56^**2b**,^57^ and **2c**(58) in high yields (83, 80, and 87%, respectively) after purification by column chromatography followed by recrystallization (detailed synthetic procedures and characterization are provided in the Supporting Information). The structure of the three NFSMAs were verified by ^1^H and ^13^C NMR spectroscopies and MALDI-TOF mass spectrometry. The thermal stabilities of the three NFAs were assessed by thermogravimetric analysis. **FG6**, **FG8**, and **FG10** show thermal decomposition temperatures (*T*_d_) of 329, 325, and 307 °C, respectively, thus indicating their excellent thermal stabilities (Figure S16 and Table S1).

**Scheme 1:**
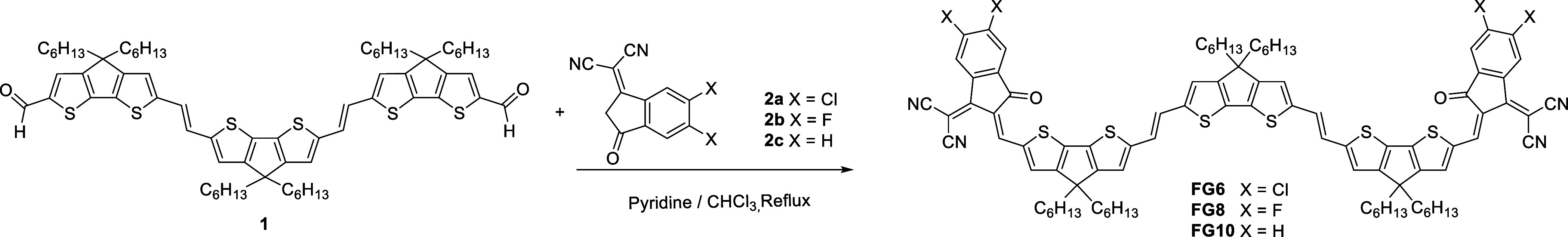
Synthesis of **FG6**, **FG8**, and **FG10**

Theoretical studies were carried out using a DFT (B3LYP) method with 6-31G basis set to evaluate the most stable conformations and frontier orbital levels of all A–D–A-type NFSMAs. The optimized molecular geometries are shown in Figures S17–S19. In an effort to reduce the calculation time, the hexyl chains were substituted by methyl units. The computations revealed that the three compounds have planar conformations with dihedral angles of 0° throughout the conjugated system. The calculated S···O (2.71 Å) and N···H (2.43 Å) distances are smaller than the sum of the van der Waals radii (3.25 and 2.74 Å, respectively), and this indicates that these intramolecular interactions between the external units and the CPDT oligomer contribute to a more planar conjugated system. The estimated HOMO/LUMO energy levels are −5.10/–3.48, −5.05/–3.42, and −4.95/–3.29 eV for **FG6**, **FG8**, and **FG10**, respectively (Scheme 1).

**Figure 1:**
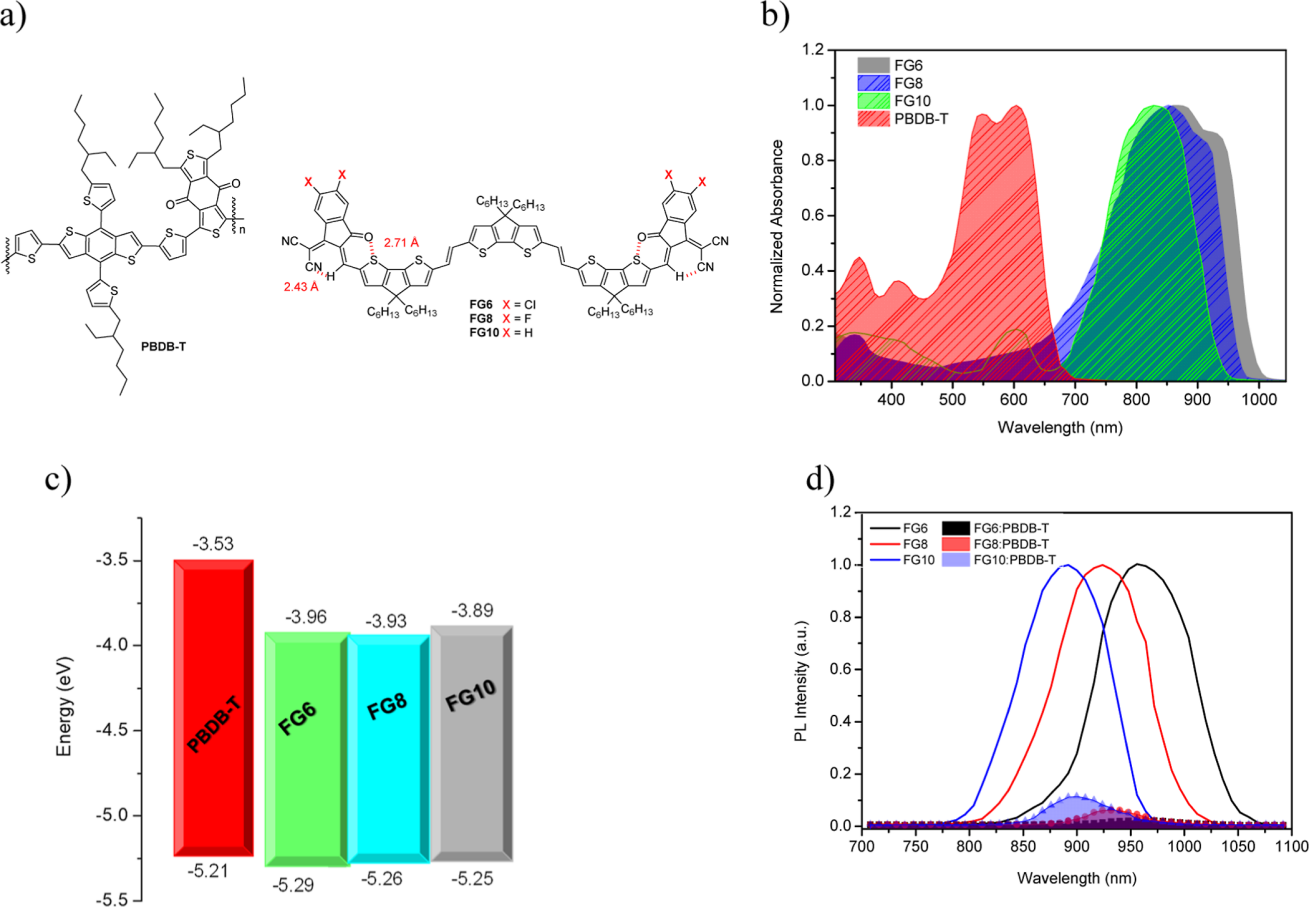
Structure (a), absorption spectra in thin film (b), frontier orbital levels calculated from electrochemical data (c) and photoluminescence (d) of PBDB-T, **FG6**, **FG8**, and **FG10**.

The absorption profiles of **FG6**, **FG8**, and **FG10** were studied in solution of CHCl_3_ (Figure S20) as well as thin films (Figure 1b), and the optical data are compiled in Table 1. In solution, all three compounds presented an absorption range between 400 and 950 nm. The absorption spectra of **FG6** and **FG8** showed red-shifts of around 30 and 10 nm, respectively, when compared with **FG10**. In comparison to chloroform solution, the thin-film spectra reveal an apparent red-shifted absorption peak (Figure 1b), with the bathochromic shift being more marked in halogenated compounds **FG6** and **FG8** owing to the inductive effect of the halogen atoms leading to better intermolecular charge transfer.^48^ Moreover, the thin-film spectra of all three compounds contained a shoulder. This shoulder was stronger for **FG6** and extended the absorption up to 1000 nm. The presence of this shoulder demonstrates stronger π–π intermolecular interactions owing to the presence of a chloro-substituent in the terminal unit and is a consequence of the stronger electron-accepting nature of chlorinated terminal groups when compared to the fluorinated counterpart.^59,60^

**Table 1:** Optical Data, Redox Properties, and Frontier Orbitals of **FG6**, **FG8**, and **FG10**

	λ_abs_/nm (log ε)^a^	λ_abs_ (nm)^b^	*E*_red_^1^ (V)^c^	*E*_ox_^1^ (V)^c^	*E*_HOMO_ (eV)	*E*_LUMO_ (eV)	*E*_gap_ (eV)
**FG6**	838 (5.87)	868	–1.14	0.19	–5.29	–3.96	1.33
		932					
**FG8**	828 (5.81)	852	–1.17	0.16	–5.26	–3.93	1.33
**FG10**	808 (5.71)	828	–1.21	0.15	–5.25	–3.89	1.36

a In chloroform (1.8 × 10^–6^ M).

b In thin films.

c See the Experimental Conditions section in the Supporting Information.

The optical band gap values for these materials were assessed from their absorption onset in the thin-film absorption profile as 1.28, 1.32, and 1.34 eV for **FG6**, **FG8**, and **FG10**, respectively. The thin-film absorption spectrum of PBDB-T is also displayed in Figure 1b, and this is complementary to those of the acceptor materials, which is beneficial for capturing more photons to achieve high *J*_SC_ values.

The HOMO and LUMO energy levels of these acceptors were determined by OSWV and cyclic voltammetry (CV) (Figures S21–S26). The HOMO/LUMO levels of **FG6**, **FG8**, and **FG10** were calculated based on *E*_red_ and *E*_ox_ values (*E*_HOMO_ = −5.1 – *E*_ox_^1^; *E*_LUMO_ = −5.1 – *E*_red_^1^) (Figure 1c, Table 1). The HOMO and LUMO energy levels of the halogenated NFSMAs showed a decreasing trend due to the stronger electron-accepting effect of the halogen atoms compared to the H atom.^61^ Despite the fact that the decrease in the LUMO level is not beneficial to achieve a high *V*_OC_, it reduces the band gap and leads to a red-shifted spectrum, which is promising for generation of exciton generation their dissociation, thus resulting in a high *J*_SC_ value.^62^ Though the chlorine atom exhibits lower electronegative than fluorine, chlorination decreases the HOMO energy level more efficiently than fluorination as the empty 3d orbitals of the chlorine atom can hold a greater electron density.^63^

The dielectric constants of the NFSMAs were measured by the impedance spectroscopy technique (detail summarized in the Supporting Information), and the values are 4.81, 4.64, and 3.78 for **FG6**, **FG8**, and **FG10**, respectively. The higher dielectric constant for **FG6** and **FG8** as compared to **FG10** leads to the larger dipole moment due to the fluorination of terminal units. The higher dielectric constant and dipole moment for **FG6** lead to a lower exciton binding energy generated in the acceptor phase after the absorption of photons. This in turn decreases the driving force (HOMO offset) required for exciton dissociation and HT from acceptor to donor.

In the BHJ–OSCs, the absorption of light leads to the exciton’s generation, and these subsequently dissociate into electrons and holes. After that, the electrons and hole are transferred from donor to acceptor and acceptor to donor, respectively. The HT and electron transfer (ET) depend on the HOMO and LUMO offset between donor and acceptor, respectively. As shown in Figure 1c, the LUMO offset for all the BHJ active layers is quite high, and this is sufficient for effective ET from the donor (PBDB-T) to the acceptors (**FG6**, **FG8**, or **FG10**). However, the HOMO offsets are quite small, and therefore, the thin-film PL spectra of pristine acceptors and their blends with PBDB-T were recorded (Figure 1d). The PL intensity of all acceptors is considerably quenched when blended with PBDB-T, and the trend in quenching is **FG6** > **FG8** > **FG10**. These results demonstrate that there is effective HT from these acceptors to PBDB-T, as observed for most NFA-based PSCs even when the HOMO offset is very small or zero, as reported in the literature.^64−66^ As discussed above, the dielectric constant for **FG10** is lower than that for the other materials, with **FG6** > **FG8** > **FG10**, and this leads to a reduced exciton binding energy. This is consistent with the highest PL quenching for PBDB-T/**FG6**.^66,67^ A low driving force is needed for dissociation of excitons generated in **FG6** and HT from **FG6** to PBDB-T.

**Figure 2:**
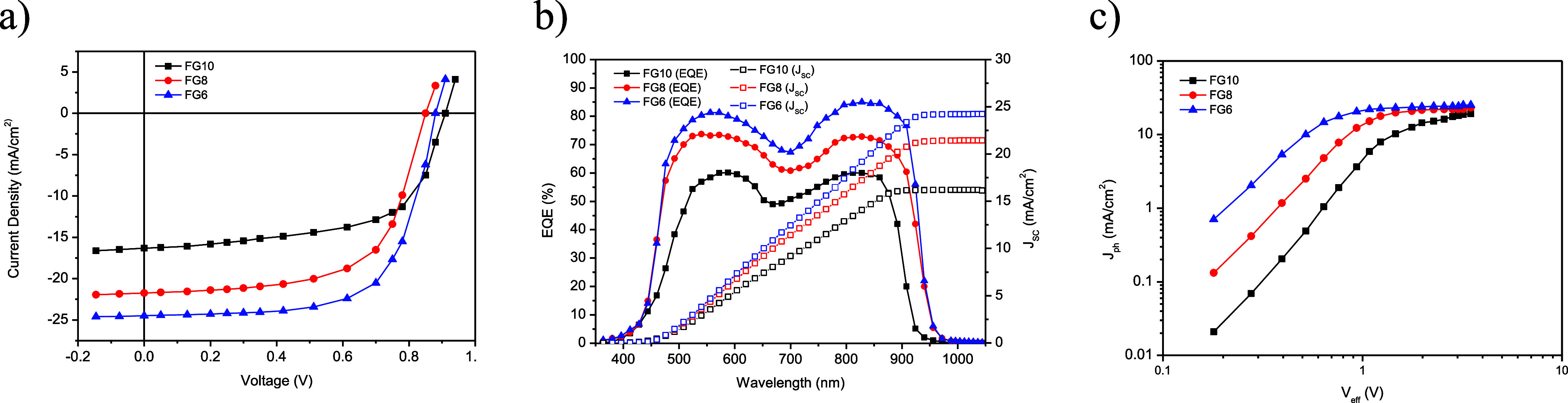
(a) *J*–*V* plots and (b) EQE and *J*_SC_ spectra of the binary systems (PBDB-T/**FG6**, PBDB-T/**FG8**, and PBDB-T/**FG10**); and (c) dependence of *J*_ph_ with *V*_eff_.

The BHJ–OSC devices were fabricated with the conventional ITO/PEDOT/PSS/active layer/PFN design. The description of device fabrication and their photovoltaic characterization is summarized in the Supporting Information. Initially, the weight ratios between PBDB-T and acceptors (**FG6**, **FG8**, and **FG10**) were varied to optimize the photovoltaic performance of OSCs (Tables S2–S4), and the optimized ratio for all blends was 1:1.2. The next step involved a combined treatment of solvent additive (DIO) and subsequent solvent vapor annealing (SVA) using THF to optimize the photovoltaic performance of the OSCs. On using the solvent additive, the PCE of the OSCs was enhanced, but the improvement was modest. As a result, a combined treatment of solvent additive and subsequent SVA treatment was adopted. The current–voltage (*J*–*V*) plots for the optimized OSC under illumination are displayed in Figure 2a, and the corresponding photovoltaic results are collected in Table 2.

**Table 2:** Photovoltaic Data of the OSCs Prepared with Optimized Active Layers: PBDB-T/**FG6**, PBDB-T/**FG8**, and PBDB-T/**F10**

acceptor	*J*_SC_ (mA/cm^2^)	*V*_OC_ (V)	FF	PCE (%)	*E*_loss_ (eV)
**FG6**	24.48 (24.24)^a^	0.88	0.70	15.08 (14.92)^b^	0.45
**FG8**	21.74 (21.43)^a^	0.85	0.68	12.57 (12.36)^b^	0.48
**FG10**	16.28 (16.14)^a^	0.91	0.61	9.04 (8.91)^b^	0.50

a Estimated from the EQE spectra.

b Average of eight devices.

The *V*_OC_ values for devices made with **FG8** and **FG6** are lower than those for the **FG10**-based device (Figure 2a). This finding can be attributed to the electron-withdrawing effect of the halogen atoms and is coherent with the upshifted LUMO energy levels of **FG10** as compared to **FG6** and **FG8** since the *V*_OC_ value for OSCs is proportional to energy difference LUMO_acceptor_ – HOMO_donor_. Conversely, the *J*_SC_ and FF values for the devices based on **FG6** and **FG8** are higher than those for **FG10**. This difference is due to the decrease in band gap and increase in the light harvesting efficacy, exciton generation, and effective charge transfer. It can be seen from Figure 1c that the HOMO offset between PBDB-T and **FG10** is smaller when compared to those for **FG6**/PBDB-T and **FG8**/PBDB-T. As in the case of OSCs based on NFAs, photocurrent generation is due to exciton generation and the subsequent dissociation and ET from donor to acceptor and HT from acceptor to donor. In the case of PBDB-T/**FG10**, the excitons generated in **FG10** dissociate to give free charge carriers, but the holes are not able to transfer from **FG10** to PBDB-T, and this leads to the low *J*_SC_ value for **FG10**-based OSCs. The dielectric constants of the acceptors studied here are in the order **FG6** > **FG8** > **FG10**, i.e., highest for **FG6** and lowest for **FG10**. This trend will lead to a lower exciton binding energy for **FG10**, and this is beneficial for exciton dissociation and charge transfer. The same trend is observed for the *J*_SC_ values of the acceptors. Consequently, OSCs made with **FG6**, **FG8**, and **FG10** gave overall PCE values of 15.08, 12.57, and 9.04%, respectively.

External quantum efficiency (EQE) spectra of the OSCs were recorded to validate the differences in the *J*_SC_ values (see Figure 2b). In comparison to **FG10**, the value of EQEs for **FG8**- and **FG6**-based OSCs is higher over the whole wavelength range of 400–980 nm and also exhibits wider EQE response. The EQE of the device based on **FG6** is better than that for the **FG8** counterpart, and this is consistent with its higher *J*_SC_. The *J*_SC_ values obtained by integration of the EQE spectra are 24.24, 21.43, and 16.14 mA/cm^2^ for **FG6**, **FG8**, and **FG10**, respectively. These values fit quite well with the *J*_SC_ values obtained from the *J*–*V* curves.

The PCE value for an OSC is dictated by the degree of exciton generation, the dissociation into free charge carriers, and the subsequent charge transport toward the electrodes. The knowledge about the exciton generation rate, exciton dissociation probability, and charge collection probability in these devices was obtained by examining the dependence of photocurrent density (*J*_ph_) with effective voltage (*V*_eff_) for these devices (Figure 2c). The *J*_ph_ and *V*_eff_ values were estimated as described in the Supporting Information. It can be seen from the plots that *J*_ph_ initially increased in a linear manner with *V*_eff_. However, at higher *V*_eff_, the *J*_ph_ value was independent of *V*_eff_, and it then reached the saturation value (*J*_sat_), after which *J*_ph_ was only dependent on the absorption profile of the active layer. The maximum exciton generation rate (*G*_max_) can be assessed by *G*_max_ = *J*_sat_/*qL*, where *q* is the elementary charge and *L* is the thickness of the active layer. The *G*_max_ values for devices fabricated with **FG10**, **FG8**, and **FG6** are 1.24 × 10^28^, 1.51 × 10^28^, and 1.66 × 10^28^ m^–3^ s^–1^, respectively. The trend in the value of *G*_max_ indicates that the highest and lowest excitons are generated for the **FG6**- and **FG10**-based active layers, respectively. The exciton dissociation probability (*P*_diss_) and charge collection probability can be assessed from the ratio *J*_ph_/*J*_sat_ under short circuit and maximum power point conditions, respectively. The *P*_diss_/*P*_coll_ values are 0.863/0.686, 0.943/0.764, and 0.972/0.803 for **FG10**, **FG8**, and **FG6**, respectively. The highest *P*_diss_ and *P*_coll_ values were obtained for the **FG6**-based OSC and is because this compound gives rise to the highest dielectric constant and lowest exciton binding energy for the **FG6**/PBDB-T active layer. This system requires the lowest driving force for exciton dissociation and charge transfer, and this is beneficial for the enhancement of the *J*_SC_ and FF.

**Figure 3:**
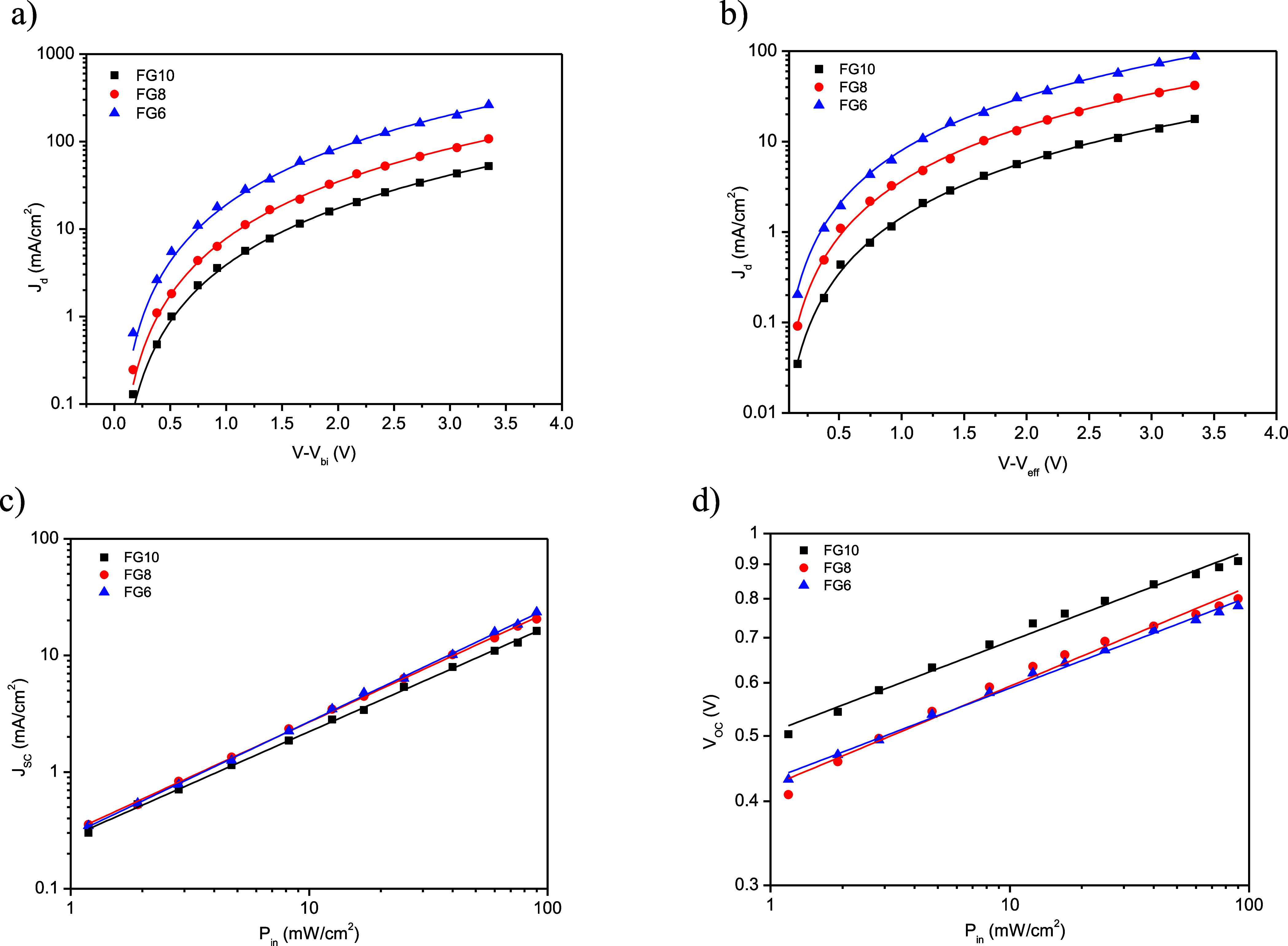
(a) Dark *J*–*V* characteristics and SCLC fitting for (a) hole only, (b) electron-only devices, dependence of (c) *J*_SC_ and (d) with *P*_in_.

The charge-transport properties in these devices were evaluated by measuring the dark *J*–*V* characteristics and fitting them with the space charge limited current (SCLC) model. Hole-only and electron-only devices were fabricated to measure the hole mobility (μ_h_) and electron mobility (μ_e_), as described in the Supporting Information. The dark *J*–*V* curves for hole and electron only devices and modeling with SCLC are shown in Figure 3a,b, respectively. The μ_h_ values for **FG6**, **FG8**, and **FG10** are 3.12 × 10^–4^, 3.02 × 10^–4^, and 2.94 × 10^–4^ cm^2^/V s, respectively. The μ_e_ values for **FG6**, **FG8**, and **FG10** are 2.51 × 10^–4^, 2.22 × 10^–4^, and 2.02 × 10^–4^, respectively. The μ_h_ and μ_e_ values for **FG6** are the highest and the μ_h_/μ_e_ ratio (1.24) is the lowest, whereas for **FG10**, the corresponding values are the lowest with the highest μ_h_/μ_e_ ratio (1.46). This finding indicates that charge transport is most balanced in the device based on **FG6**/PBDB-T and least balanced in the **FG10**/PBDB-T device, thus resulting in the highest and lowest FF values for the former and latter devices, respectively (Table 2).

The charge recombination processes in the devices were analyzed by the change of *J*_SC_ (Figure 3c) and *V*_OC_ (Figure 3d) with illumination intensity (*P*_in_).

The dependence of *J*_SC_ with *P*_in_ (Figure 3c) follows the power law *J*_SC_ ∝ (*P*_in_)^α^, where α is an exponential factor that provides evidence about the degree of bimolecular charge recombination in the devices. The values of α for the PBDB-T/**FG6**, PBDB-T/**FG8**, and PBDB-T/**FG10** devices are 0.984, 0.952, and 0.893, respectively. Ideally, the α value will be unity when bimolecular recombination is almost insignificant. If the value of α is lower than unity, it represents the degree of bimolecular recombination. The value of α for **FG6**/PBDB-T approaches unity, and this indicates that the bimolecular recombination is very efficiently suppressed when compared to **FG8**/PBDB-T and **FG10**/PBDB-T. The variation of *V*_OC_ with *P*_in_ is described by *V*_OC_ = (*nkT*/*q*)ln (*P*_in_), where *n* is the diode quality factor, *k* is Boltzmann’s constant, and *T* is the absolute temperature (Figure 3d). The value of *n* provides assessment about the degree of trap-assisted recombination. In general, if the value of *n* is around unity, then the trap-assisted recombination is almost negligible, and in cases where *n* is greater than unity, trap-assisted recombination occurs. The *n* values for PBDB-T/**FG10**, PBDB-T/**FG8**, and PBDB-T/**FG6** are 1.48, 1.36, and 1.27, respectively. The lowest *n* value was obtained for PBDB-T/**FG6**, and this indicates that the trap-assisted recombination is reduced in the PBDB-T/**FG6**-based OSCs.

The energy loss (*E*_loss_) in the OSCs plays a critical role. The *E*_loss_ values were estimated as *E*_loss_ = *E*_g_ – *qV*_OC_, where *E*_g_ is measured from the onset of the EQE spectra of the OSCs. The *E*_loss_ values for OSCs based on **FG6**, **FG8**, and **FG10** are 0.45, 0.48, and 0.50 eV, respectively. The lowest *E*_loss_ value was obtained for the **FG6**-based device, and this may be a consequence of the high dielectric constant of **FG6**, which reduces the binding energy of exciton and lowers the driving force required to separate the free charge carrier from the exciton. This in turn leads to efficient HT from **FG6** to the donor PBDB-T when compared to the other devices and leads to a reduction in the non-radiative energy loss. The PL intensity of **FG6** is quenched more for **FG6**/PBDB-T as compared to other blends, which also validate the lowest non-radiative loss in the **FG6**-based OSCs.

We have also determined the dielectric constant of neat compounds and blends (Figures S27 and S28), and the values are about 4.7, 4.4, and 3.6 for **FG6**/PBDB-T, **FG8**/PBDB-T, and **FG10**/PBDB-T blends, respectively. The higher value of the dielectric constant for **FG6** based blend also indicates that the exciton binding energy is reduced in this blend as compared to other blends. The reduced exciton binding energy also indicates that the less driving force is needed for both ET and HT from donor to acceptor and acceptor to hole, respectively, leading to reduced non-radiative energy loss for the **FG6**-based OSCs.

**Figure 4:**
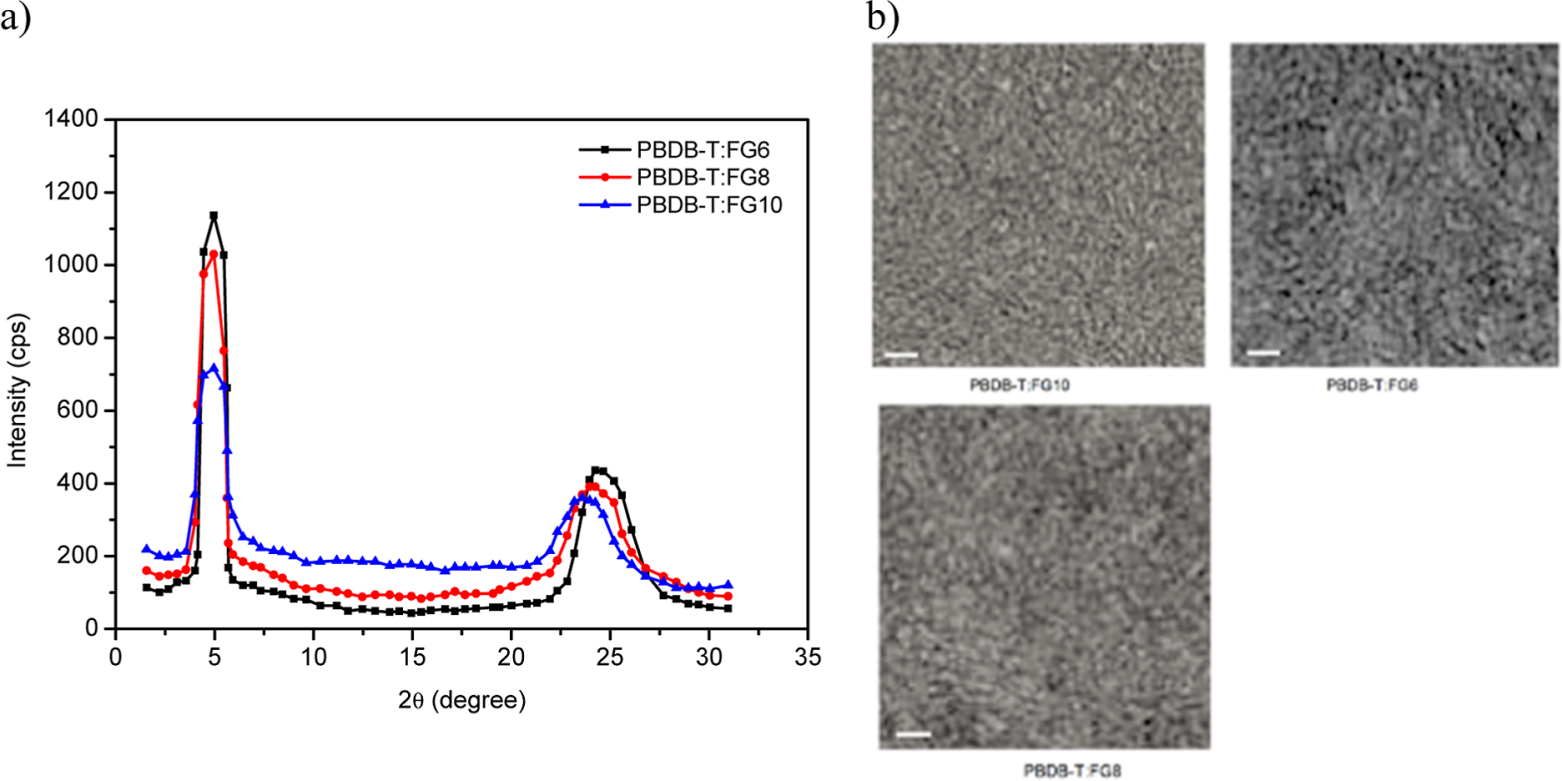
(a) XRD patterns and (b) TEM images of PBDB-T/**FG6**, PBDB-T/**FG8**, and PBDB-T/**FG10** films (scale bar is 100 nm).

The X-ray diffraction (XRD) patterns of thin films of the pristine acceptors are shown in Figure S29. The patterns of the pristine **FG6** and **FG8** films display π–π stacking diffraction peaks at 2θ = 24.45° and 2θ = 24.12°, respectively, while for **FG10**, the corresponding peak is located at 2θ = 23.89°. These peaks indicate that the π–π stacking distances follow the order **FG6** < **FG8** < **FG10**. These results suggest that halogenation of the terminal units has a considerable impact on the molecular crystallinity and π–π stacking distance. In all acceptors, the lamellar diffraction (100) is 2θ = 5.12° and the intensity varies in the order **FG6** > **FG8** > **FG10**, whereas different (010) π–π stacking diffraction peaks are observed at 2θ = 24.65, 24.24, and 23.86° for **FG6**, **FG8**, and **FG10**, respectively, indicating that the π–π stacking distance is reduced, and the crystallinity is increased, after the halogenation of terminal units. The PBDB-T/**FG6**, PBDB-T/**FG8**, and PBDB-T/**FG10** blend films gave rise to π–π diffraction peaks at 24.23, 23.96, and 23.58° (Figure 4a). These peaks correspond to the π–π interaction distances of 0.349, 0.357, and 0.364 nm, respectively. The crystal coherence length (CCL) for the PBDB-T/**FG6**, PBDB-T/**FG8**, and PBDB-T/**FG10** systems is 3.63, 3.42, and 3.17 nm, respectively. A small π–π stacking distance and larger CCL are beneficial for efficient charge transport and a reduction in the recombination. The lowest and highest CCL values were obtained for PBDB-T/**FG10** and PBDB-T/**FG6**, respectively, being consistent with the trend observed in the FF values of the OSCs. The π–π stacking distance is reduced for the blend based on the halogenated acceptor blend as compared to the nonhalogenated counterpart, indicating that the molecular packing density increased for the halogenated acceptor blend which leads to the increased dielectric constant and also helps hold the large phase separation in the BHJ active layer, through which charge recombination in the active layer can be suppressed, favorable for high FF of the OSCs.

The phase separation and morphology of the active layer are crucial for charge transport and recombination toward the electrodes impact on the FF of the OSCs. These characteristics were investigated for the materials reported here by obtaining transmission electron microscopy (TEM) images of the optimized blended films (Figure 4b). The dark and bright regions observed in the TEM images correspond to the acceptor and donor rich domains, respectively. It can be seen from the images, as shown in Figure 4b, that the morphology for PBDB-T/**FG6** is the most appropriate with enlarged interfacial area and bi-continuous pathway networks compared to the others owing to the cooperative effect between the larger spacer effect and higher electronegativity of the halogen atom. The incorporation of the halogen atom into the NFSMA boosts the intermolecular π–π interactions in the blended film, which leads to realize the well-defined orientations both morphologically and in the crystalline domains of blended films at the nanoscale. The better and well-defined phase separation for PBDB-T/**FG6**, when compared to the other blends, leads to efficient charge transport and suppressed recombination in the OSCs based on this system.^68−70^ This arrangement results in the highest *J*_SC_ and FF values of all materials reported here.

## Conclusions

Three new non-fused A–D–A NFAs have been designed, synthesized, and characterized. The molecules have the same donor core but different IC terminal units, namely, **FG10** (2H-IC), **FG8** (di-fluorinated IC), and **FG6** (di-chlorinated IC). The impact that di-halogenation of the terminal groups has on the optoelectronic and electrochemical characteristics of these acceptors and corresponding photovoltaic performance have been examined. Compared to **FG10**, both **FG6** and **FG8** showed red-shifted absorptions, and this was attributed to the inductive effects caused by the presence of halogen atoms in the IC terminal units. The XRD spectra of pristine acceptor films indicate that a smaller π–π interaction distance led to enhanced electron mobility is the solid state for the halogenated terminal acceptor. The **FG10**-based OSC gave a PCE of 9.04% with a *J*_SC_ value of 16.28 mA/cm^2^ and an FF of 0.61. These values are attributed to the larger π–π stacking distance and inadequate π–π packing arrangement. Compared to the **FG10**-based OSC, the **FG8**-based OSC gave a higher *J*_SC_ value, and this is attributed to the red-shifted and enhanced charge carrier mobility. In the case of **FG6**, the strong π–π interaction (reduced distance) yielded a low ratio between hole and electron mobility. As a result, the **FG6**-based OSC showed a remarkable PCE of 15.08% with a *J*_SC_ of 24.48 mA/cm^2^, an FF of 0.70, and an *E*_loss_ of 0.45 eV. The low *E*_loss_ value for the **FG6**-based OSC is associated with the higher dielectric constant of **FG6** when compared to **FG8** and **FG10**. This higher dielectric constant leads to a lower exciton binding energy and a lower driving force required for exciton dissociation and HT from **FG6** to PBDB-T. This situation results in a reduction in the non-radiative energy loss.

## Experimental Section

### Procedure for the Knoevenagel Condensation Reaction

This reaction was carried out according to the experimental procedure described before.^35^ The resulting solid, after evaporation of the solvent, was purified by column chromatography and eventually recrystallized by slow vapor diffusion with the specified solvents to obtain **FG6**, **FG8**, and **FG10** as pure solids.

**FG6**. Synthesis: bisaldehyde **1**(40) (75 mg, 0.066 mmol, 1 equiv), 3-(dicyanomethylidene)-5,6-dichloro-indan-1-one (**2a**)^56^ (122 mg, 0.462 mmol, 7 equiv), pyridine (330 μL), and anhydrous CHCl_3_ (6 mL). Reaction time: 5 h. Purification: column (silica-gel, hexane/CHCl_3_: 1:4) and recrystallization diffusing methanol into a solution of CHCl_3_. Yield: 83%. mp >300 °C. ^1^H NMR (400 MHz, CDCl_3_): δ/ppm 8.83 (s, 2H), 8.71 (s, 2H), 7.87 (s, 2H), 7.19 (d, 2H, *J* = 15.7 Hz), 7.00 (d, 2H, *J* = 15.7 Hz), 6.99 (m, 4H), 6.95 (s, 2H), 1.90 (m, 12H), 1.18 (m, 35H), 0.99 (m, 13H), 0.83 (m, 18H). ^13^C NMR (100 MHz, CDCl_3_): δ/ppm 186.2, 167.8, 160.2, 158.4, 158.3, 153.7, 144.2, 139.9, 139.1, 138.7, 138.1, 136.1, 135.5, 126.8, 126.7, 125.1, 124.7, 122.7, 122.6, 120.6, 119.7, 115.2, 115.1, 66.6, 54.2, 54.0, 38.0, 37.9, 32.0, 31.9, 31.8, 31.7, 29.9, 29.8, 29.7, 24.9, 24.7, 22.9, 22.8, 22.7, 14.3, 14.2, 14.1. FT-IR (ATR) ν/cm^–1^: 2949, 2924, 2852, 2214, 1688, 1571, 1531, 1480, 1374, 1326, 1270, 1220, 1127, 1011, 913, 781, 671, 593. MS (MALDI-TOF) (*m*/*z*): 1631.723 (M + H) calcd for C_93_H_94_Cl_4_N_4_O_2_S_6_, 1630.445. UV–vis (CHCl_3_) λ_max_/nm (log ε) 838 nm (5.87).

**FG8**. Synthesis: bisaldehyde **1**(40) (110 mg, 0.096 mmol, 1 equiv), 3-(dicyanomethylidene)-5,6-difluoro-indan-1-one **2b**(57) (154 mg, 0.669 mmol, 7 equiv), pyridine (494 μL), and anhydrous CHCl_3_ (8 mL). Reaction time: 7 h. Purification: column (silica-gel hexane/CHCl_3_: 1:5) and recrystallization diffusing methanol into a solution of CHCl_3_. Yield: 80%. mp >300 °C. ^1^H NMR (400 MHz, CDCl_3_): δ/ppm 8.82 (s, 2H), 8.50 (dd, 2H, *J* = 10.0 Hz, *J* = 6.5 Hz), 7.62 (t, 2H, *J* = 7.6 Hz), 7.53 (br s, 2H), 7.19 (d, 2H, *J* = 15.5 Hz), 6.99 (m, 4H), 6.95 (s, 2H), 1.90 (m, 12H), 1.18 (m, 38H), 1.00 (m, 10H), 0.83 (m, 18H). ^13^C NMR (100 MHz, CDCl_3_): δ/ppm 186.3, 167.5, 160.2, 159.7, 153.3, 152.9, 144.0, 139.4, 138.0, 137.8, 136.6, 135.4, 134.5, 125.0, 122.5, 120.5, 119.8, 118.6, 115.2, 115.1, 114.8, 112.2, 66.8, 54.0, 38.0, 37.9, 31.8, 31.7, 29.8, 29.7, 24.7, 22.9, 22.8, 22.7, 14.3, 14.2, 14.1. FT-IR (ATR) ν/cm^–1^: 2951, 2923, 2852, 2214, 1689, 1603, 1586, 1533, 1482, 1378, 1267, 1211, 1126, 1093, 981, 910, 602. MS (MALDI-TOF) (*m*/*z*): 1566.024 (M + H) calcd for C_93_H_94_F_4_N_4_O_2_S_6_, 1566.563. UV–vis (CHCl_3_) λ_max_/nm (log ε) 828 nm (5.81).

**FG10**. Synthesis: bisaldehyde **1**(40) (70 mg, 0.061 mmol, 1 equiv), 3-(dicyanomethylidene)-indan-1-one **2c**(58) (83 mg, 0.428 mmol, 7 equiv), pyridine (305 μL), and anhydrous CHCl_3_ (6 mL). Reaction time: 24 h. Purification: column (silica-gel, hexane/CHCl_3_: 1:2). Yield: 87%. mp 278–279 °C. ^1^H NMR (400 MHz, CDCl_3_): δ/ppm 8.83 (s, 2H), 8.64 (d, 2H, *J* = 7.2 Hz), 7.88 (d, 2H, *J* = 7.2 Hz), 7.73 (m, 2H), 7.55 (br s, 2H), 7.16 (d, 2H, *J* = 15.4 Hz), 6.98 (m, 4H), 1.91 (m, 12H), 1.19 (m, 36H), 1.01 (m, 12H), 0.83 (m, 18H). ^13^C NMR (100 MHz, CDCl_3_): δ/ppm 188.7, 166.8, 165.6, 160.8, 160.1, 152.4, 144.0, 140.1, 139.5, 136.9, 135.4, 134.2, 134.1, 129.9, 125.1, 123.4, 119.9, 63.2, 54.0, 38.1, 37.9, 31.8, 31.7, 29.8, 29.7, 24.8, 22.8, 14.2. FT-IR (ATR) ν/cm^–1^: 2950, 2922, 2852, 2213, 1690, 1602, 1586, 1532, 1481, 1377, 1350, 1269, 1229, 1211, 1126, 1094, 979, 910, 889, 723, 601, 565, 538, 419. MS (MALDI-TOF) (*m*/*z*): 1494.458 (M + H) calcd for C_93_H_98_N_4_O_2_S_6_, 1494.601. UV–vis (CHCl_3_) λ_max_/nm (log ε) 808 nm (5.71).

## Supporting Information Available

Experimental details, structural characterization spectra, electrochemical data, theoretical calculations, and details of the fabrication of OSCs and their characterization (PDF)

## Dedication

Dedicated to Professor Tomás Torres on the occasion of his 70th birthday.
