# Supplementary material for: Effects of Halogenation on Cyclopentadithiophenevinylene-Based Acceptors with Excellent Responses in Binary Organic Solar Cells
Source: ACS Appl Mater Interfaces. 2023 Apr 19;15(17):21296–305. doi: 10.1021/acsami.3c01487 (PMC11165453; doi:10.1021/acsami.3c01487)
Supplement: Supplementary file 1 — am3c01487_si_001.pdf [file am3c01487_si_001.pdf]

Supporting Information

Effects of halogenation on cyclopentadithiophenevinylene-based acceptors with excellent responses in binary organic solar cells

Fernando G. Guijarro,[a,†] Pilar de la Cruz,[,a] Kanupriya Khandelwal,[b] Rahul Singhal[c], Fernando Langa,[*,a] and Ganesh D. Sharma,[*,b,d]*

[a] Universidad de Castilla-La Mancha, Instituto de Nanociencia, Nanotecnología y Materiales Moleculares (INAMOL), Campus de la Fábrica de Armas, 45071-Toledo, Spain.
E-mail: Fernando.Langa@uclm.es, pilar.cruz@uclm.es

[b] Department of Physics, The LNM Institute of Information Technology, Jamdoli, Jaipur (Rai), 302031, India.
E-mail: gdsharma273@gmail.com and gdsharma@lnmiit.ac.in

[c]Department of Physics, Malviya National Institute of Technology, JLN Marg, Jaipur (Raj.) 302017, India

[d]Department of Electronics and Communication Engineering, The LNM Institute of Information Technology, Jamdoli, Jaipur (Rai), 302031, India.
† present adress: Departamento de Química Orgánica. Universidad Autónoma de Madrid. 28049-Madrid, Spain.

CONTENTS:

1. **General Remarks.** 1
2. **^{1}H NMR, ^{13}C NMR, FT-IR, MALDI-TOF spectra and HPLC profiles.** 4
4. **Theoretical calculations.** 12
5. **Absorption spectra in solution.** 13
6. **Electrochemical Studies.** 13
7. **XRD data** 16
8. **Photovoltaic data** 17

1. General Remarks.

Experimental conditions. Solvents and chemicals were purchased from Aldrich Chemicals (Milwaukee, WI). Anhydrous solvents, where indicated, were dried using a

Pure-Sov 400 or using standard techniques. Chromatographic purifications were performed using silica gel 60 VWR (particle size 0.040-0.063 mm). Analytical thin-layer chromatography was performed using Merck TLC silica gel 60 F254. ^{1}H NMR spectra were recorded as solutions in a partial deuterated solvent on a Brüker-Topspin AV 400 instrument. Chemical shifts are given as δ values. ^{1}H NMR chemical shifts are reported relative to residual non deuterated solvent peaks. ^{13}C NMR chemical shifts are reported relative to the deuterated solvent peak. Fourier transform infrared spectrophotometer (FT-IR) Jasco FT/IR-6800 spectrometer was used ATR (Attenuated Total Reflection) method, in each case the most characteristic bands are indicated for each compound. MALDI-TOF spectra were obtained in a Bruker UltrafleXtreme mass spectrometer, using dithranol [1,8-dihydroxy-9(10H)-anthracenone] as matrix. Analytical HPLC profiles were recorded using Agilent 1260 (column: Buckyprep (4.6ID x 250 mm)). UV/Vis spectra were recorded on a Shimadzu UV-VIS-NIR spectrophotometer UV-3600 in quartz cuvettes with a path length of 1 cm. The films for the UV-VIS-NIR absorption measurements were prepared by spin-coating a solution of the respective small molecule in chloroform on a glass substrate. Cyclic and Oyster-Young Square wave voltammetry were performed in a µAUTOLAB Type II potentiostat, using 0.1M solution of Tetrabutylammonium perchlorate in 1,2-dichlorobenzene:acetonitrile 4:1 as a supporting electrolite. Solutions were deoxygenated by bubbling argon through prior to each measurement which was run under an argon atmosphere. Experiments were carried out in a one-compartment cell equipped with a glassy carbon electrode, a platinum wire counter electrode and an $Ag/AgNO_3$ as pseudo reference electrode. All Potentials were checked against the ferrocene/ferrocenium couple (Fc/Fc+) after each experiment. Melting points are recorded in Gallenkamp melting point apparatus and are uncorrected. Thermogravimetric analyses were performed using a TGA/DSC Linea Excellent instrument by Mettler-Toledo and collected under inert atmosphere of nitrogen with a scan rate of 10 °C min^{-1}. The weight changes were recorded as a function of temperature. Theoretical calculations were carried out within the density functional theory (DFT) framework by using the Gaussian 09, applying density functional theory at the B3LYP level. The basis set of 6-31G* was used in the calculations (Supercomputation Service of UCLM).

Preparation of devices. The BHJ organic solar cells were fabricated using the glass/ITO/ PEDOT:PSS/PBDB-T:**FG6**, **FG8** or **FG10** with different weight ratios/Al device

architecture. The indium tin oxide (ITO) patterned substrates were cleaned by ultrasonic treatment in aqueous detergent, deionized water, isopropyl alcohol, and acetone sequentially, and finally dried under ambient conditions. The anode consisted of glass substrates percolated with ITO, modified by spin coating with a PEDOT:PSS layer (40 nm) as a hole transport layer and heated for 10 min at 100° C. Mixtures of PBDB-T and **FG6** or **FG8** or **FG10** (total concentration of 16 mg/mL) with weight ratios of 1:0.4, 1:0.8, 1:1.2 and 1:1.4 in toluene as solvent were prepared and then spin cast onto the PEDOT:PSS layer and dried in an ambient atmosphere. For the optimization, PBDB-T:**FG6**, :**FG8** or :**FG10** (1:1.2) blends, processed with 3 % v/v of DIO in toluene mixture, were deposited onto the top of PEDOT:PSS and subsequent solvent vapor annealing using THF were carried out. A thin film of PFN-Br (40 nm) was deposited from the 1 mg/mL solution in methanol. The approximate thickness of the active layers was 90 ± nm. Finally, the aluminum (Al) top electrode was thermally deposited on the active layer at a vacuum of 10^{-5} Torr through a shadow mask area of 0.16 cm^2. All devices were fabricated and tested in an ambient atmosphere without encapsulation. The hole-only and electron-only devices with ITO/PEDOT:PSS/active layer /Au and ITO/Al/ active layer/Al architectures were also fabricated in a similar way, to measure the hole and electron mobility. We have measured the dark J-V characteristics and fitted with the space charge limited current model. The current–voltage (J–V) characteristics of the BHJ organic solar cells were measured using a computer controlled Keithley 2400 source meter in the dark and under a simulated AM 1.5G illumination of 100 mW/cm^2. A xenon light source coupled with the optical filter was used to give the stimulated irradiance at the surface of the devices. The EQE spectra of the devices was recorded using EQE system (Bentham make).

The dielectric constant of the acceptor materials were determined by the capacitance - voltage (C-V) measurements using the device structure ITO/PEDOT:PSS/acceptor layer/Al. C-V measurement of the devices was performed using impedance analyzer at different frequencies from 1 mHz and 100 MHz and -0.8 V DC biasing is applied. The dielectric constant (ε_r) was estimated using the expression: $\varepsilon_r = C\, d/\varepsilon_o A$, where C is the geometric capacitance of the device, d is the thickness of the film, ε_o is the permeability of vacuum and A is the area of the device.

2. ^{1}H NMR, ^{13}C NMR, FT-IR, MALDI-TOF spectra and HPLC profiles.

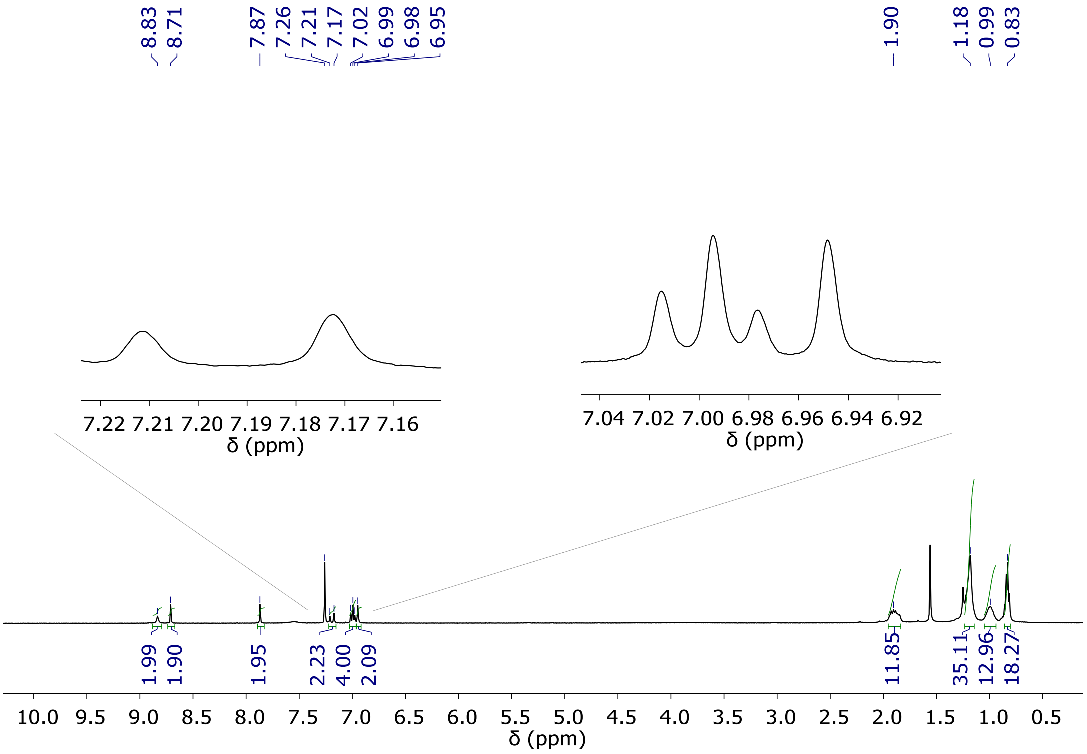

Figure S1. ^{1}H NMR (400 MHz, $CDCl_3$) spectrum of compound **FG6**

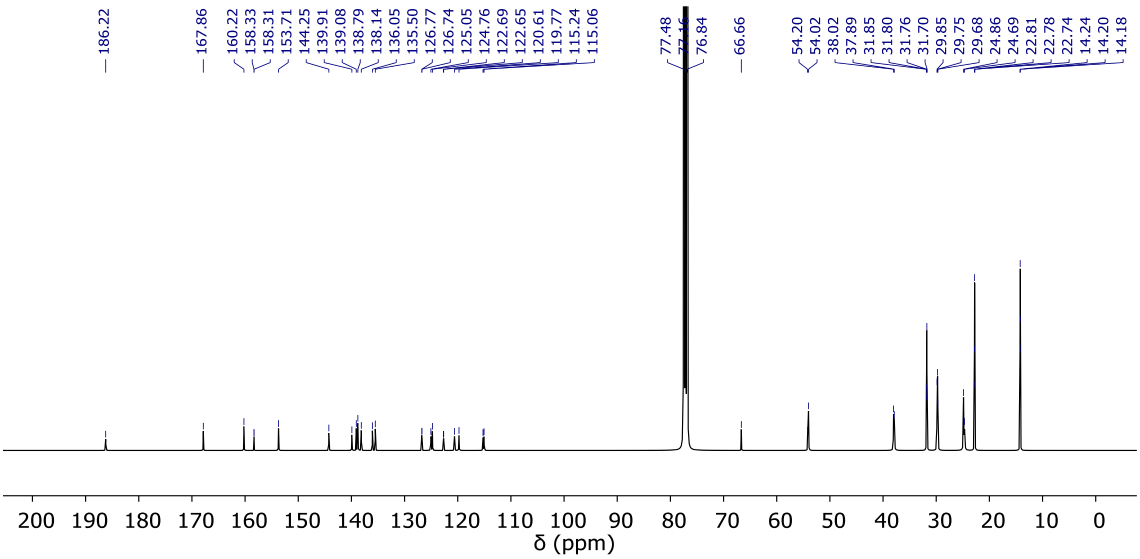

Figure S2. ^{13}C NMR (100 MHz, $CDCl_3$) spectrum of compound **FG6**

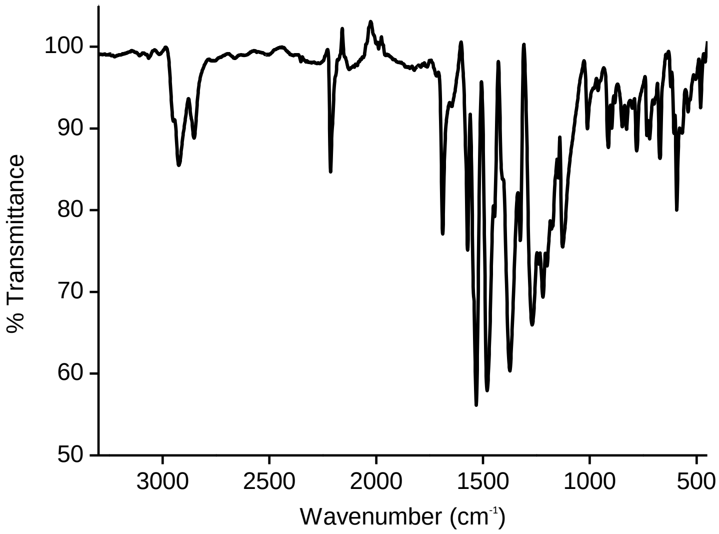

Figure S3. FT-IR (KBr) spectrum of small molecule **FG6**

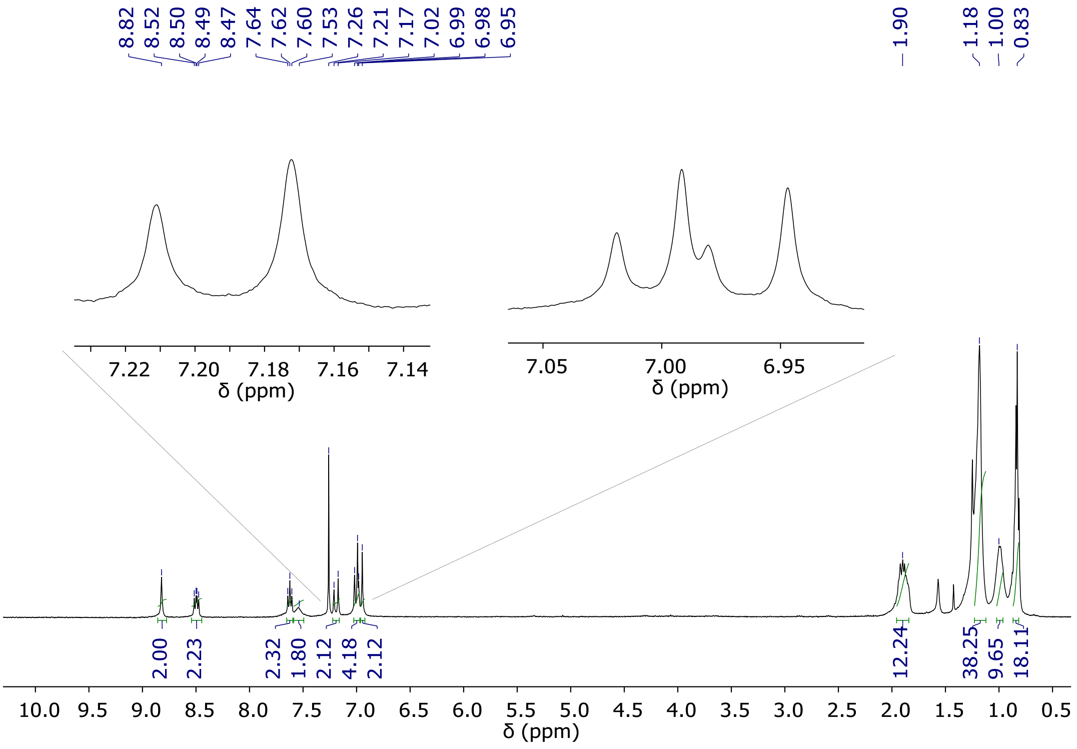

Figure S4. ^{1}H NMR (400 MHz, $CDCl_3$) spectrum of compound **FG8**

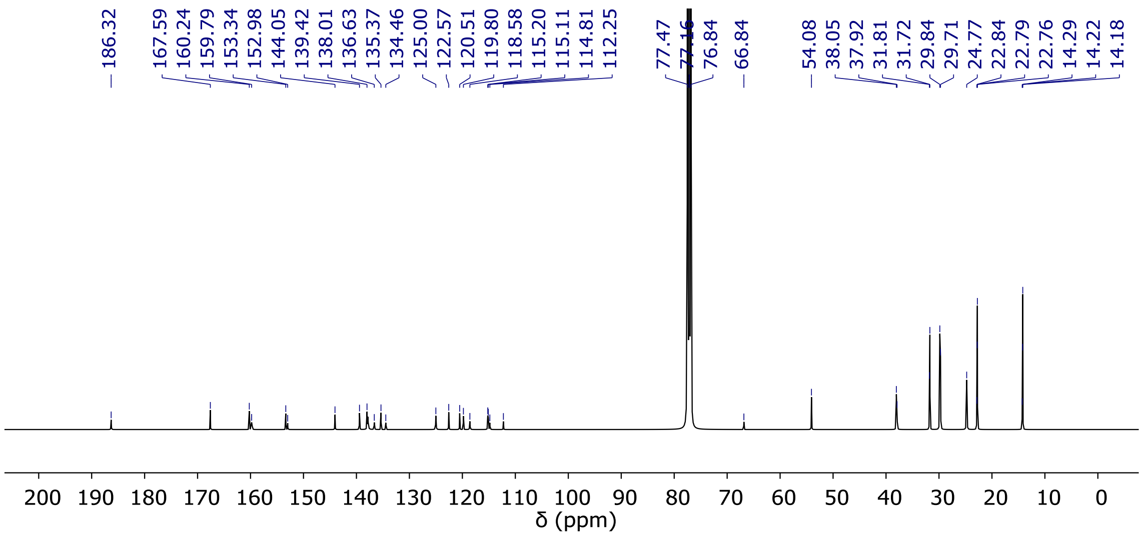

Figure S5. ^{13}C NMR (100 MHz, $CDCl_3$) spectrum of compound **FG8**

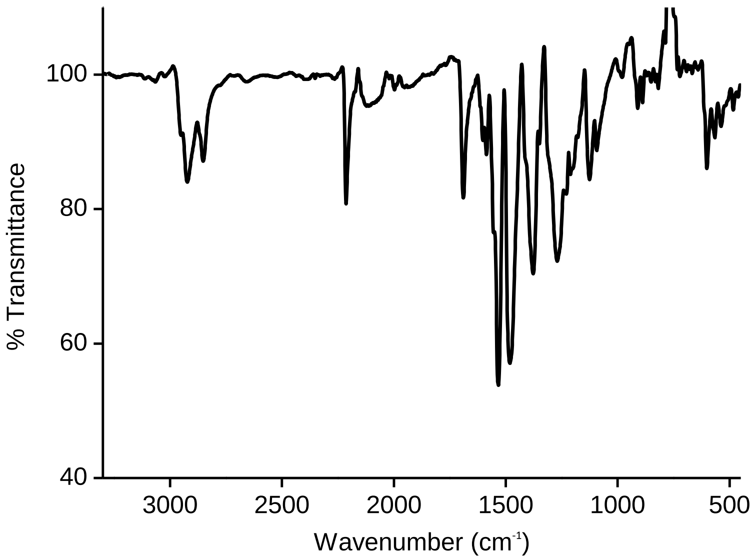

Figure S6. FT-IR (KBr) spectrum of small molecule **FG8**

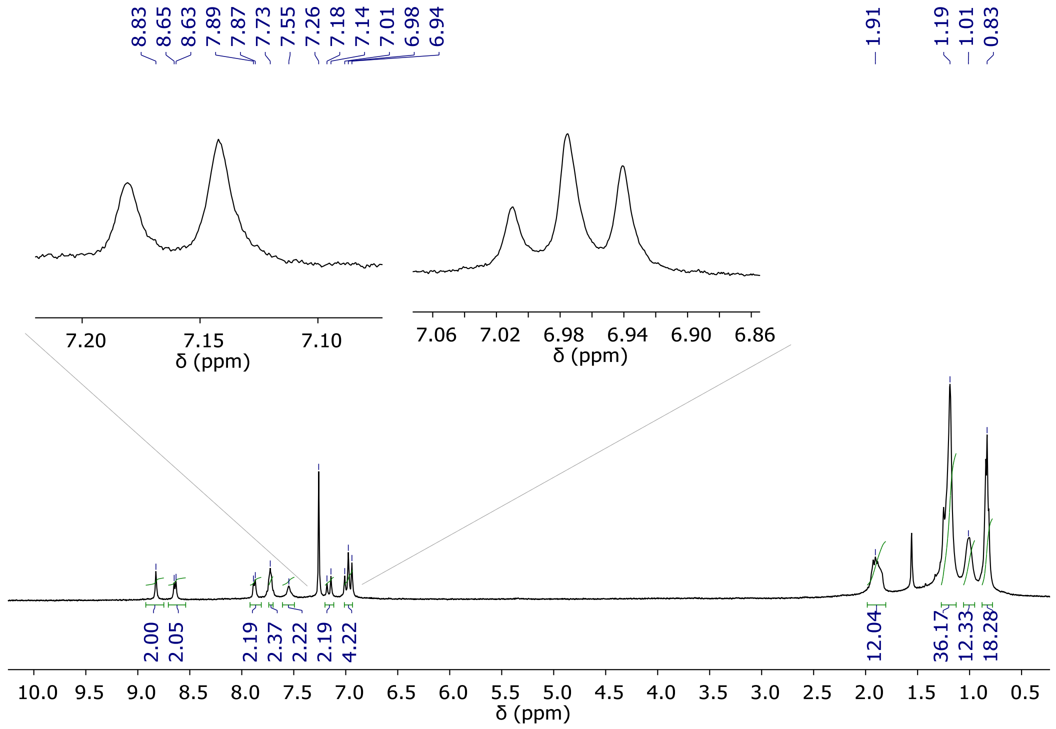

Figure S7. ^{1}H NMR (400 MHz, $CDCl_3$) spectrum of compound **FG10**

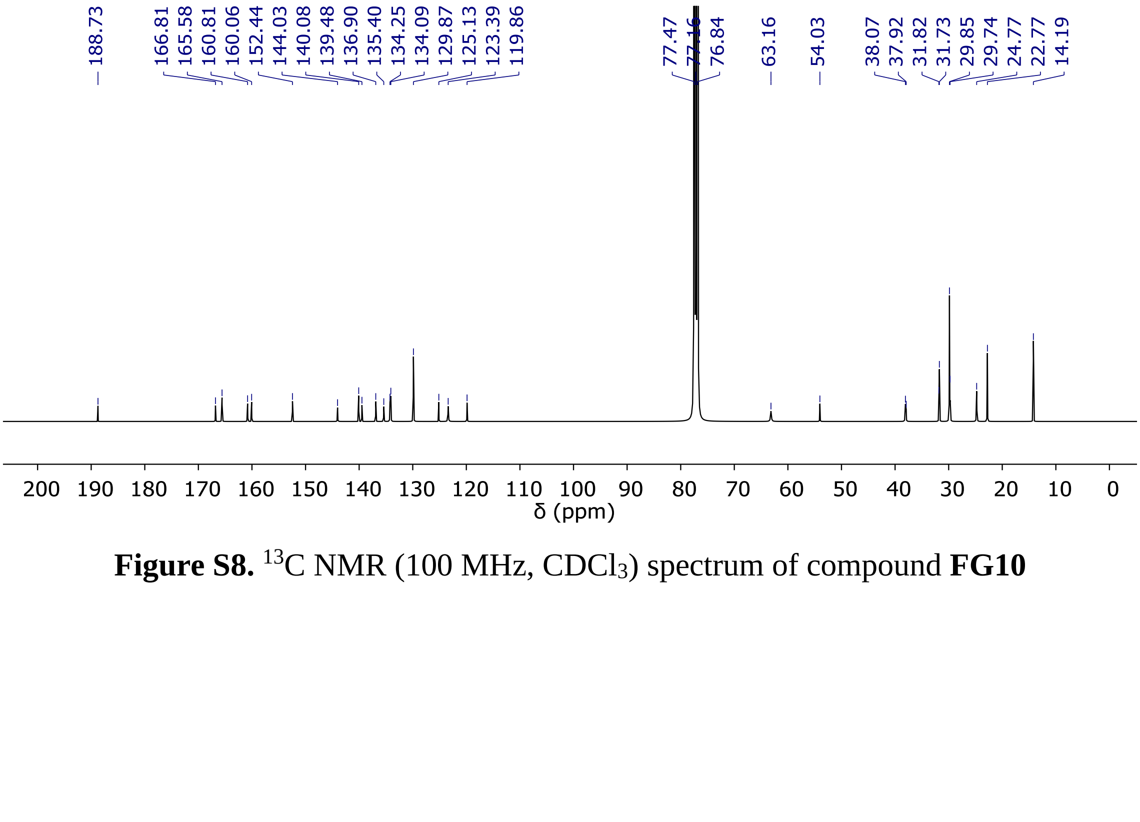

Figure S8. ^{13}C NMR (100 MHz, $CDCl_3$) spectrum of compound **FG10**

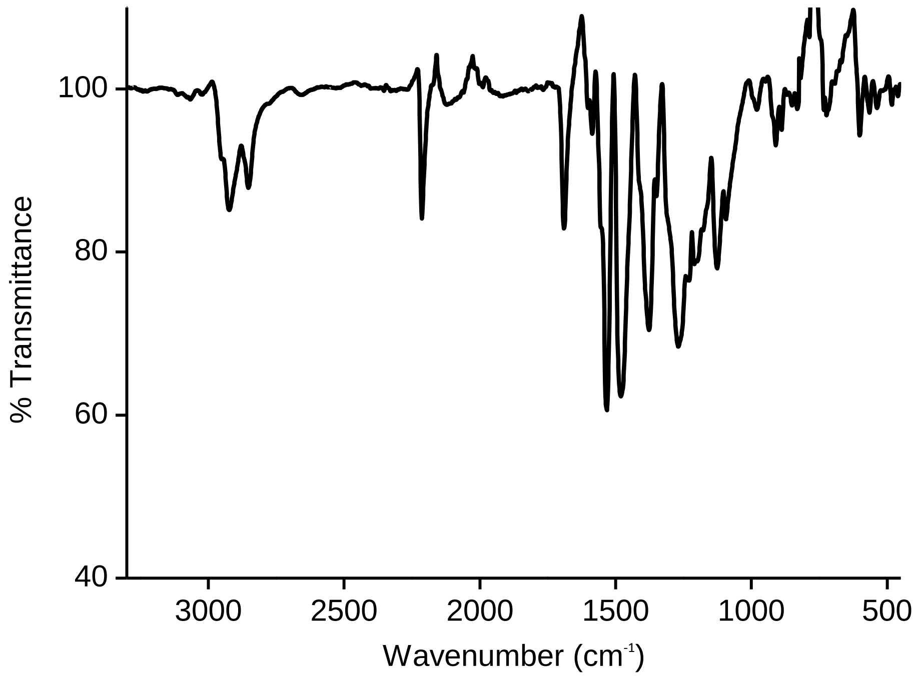

Figure S9. FT-IR (KBr) spectrum of small molecule **FG10**

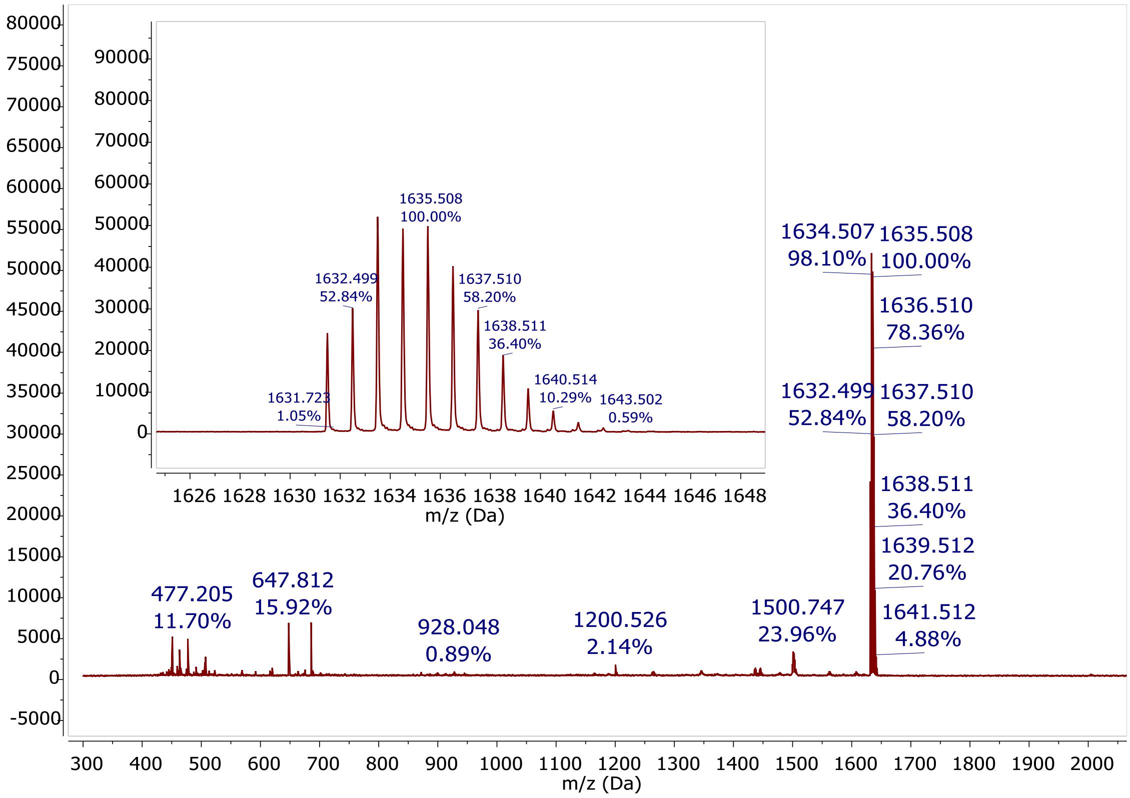

Figure S10. MS (MALDI-TOF) spectrum of compound **FG6**

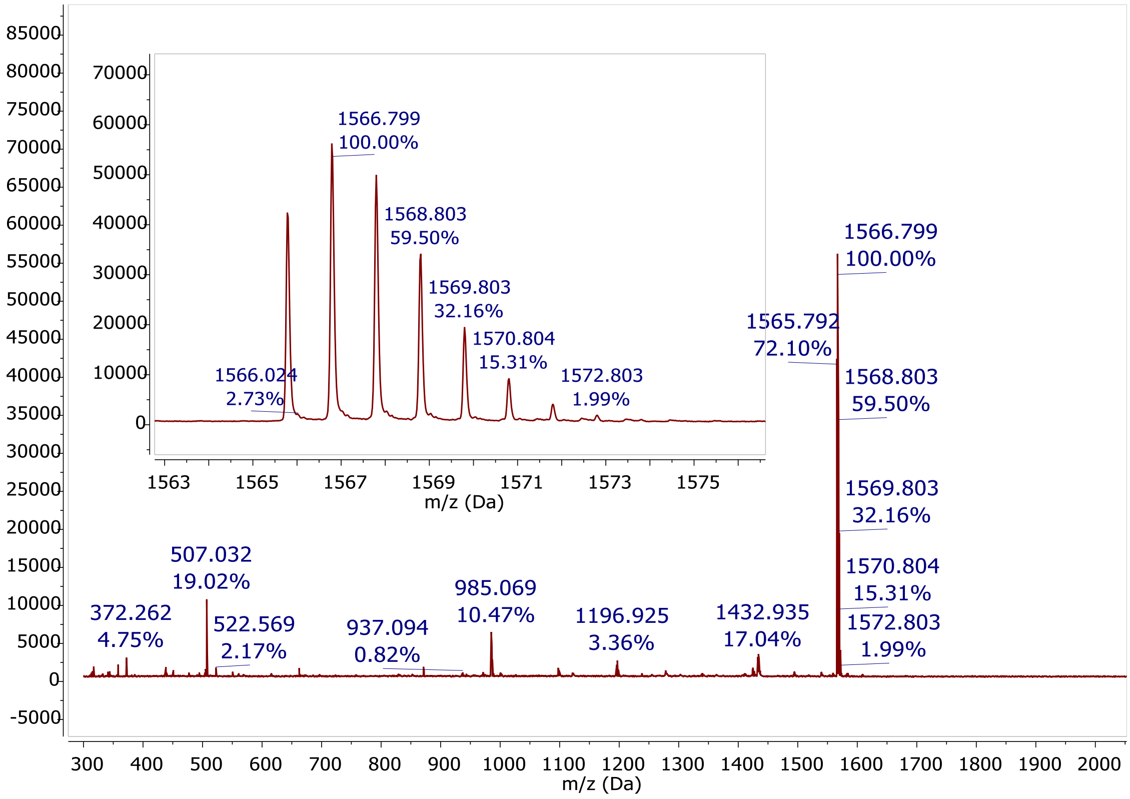

Figure S11. MS (MALDI-TOF) spectrum of compound **FG8**

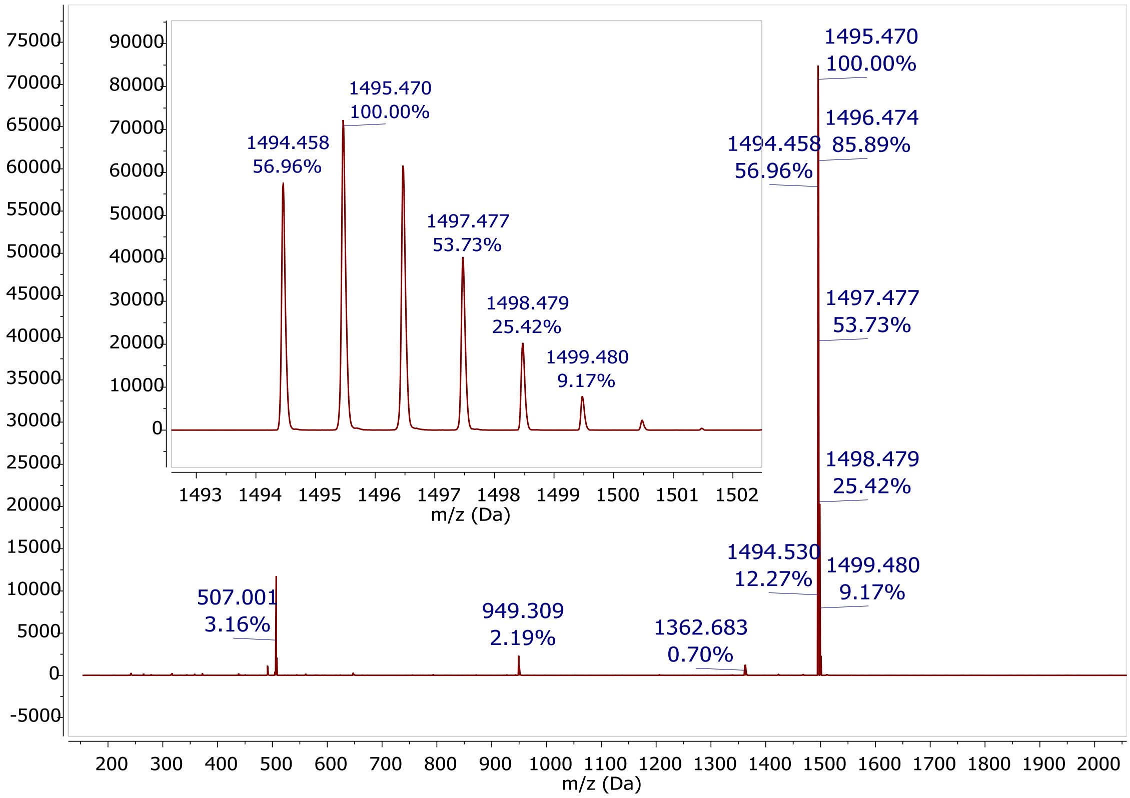

Figure S12. MS (MALDI-TOF) spectrum of compound **FG10**

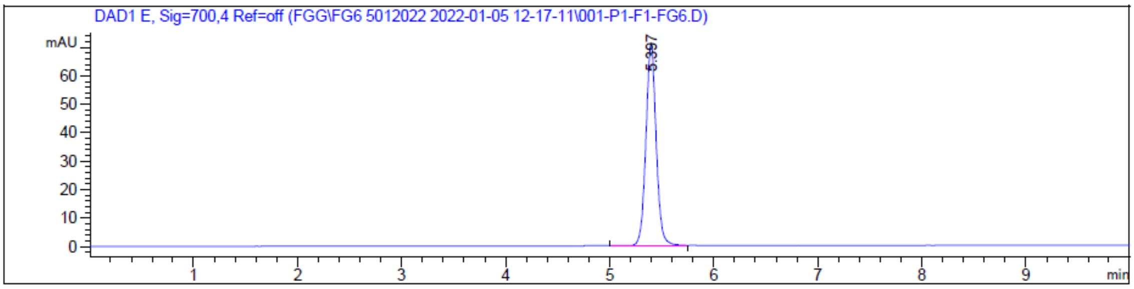

Figure S13. HPLC profile of compound **FG6**. Conditions: HPLC column: Buckyprep (4.6ID x 250 mm)), Toluene 100% as eluent (1mL/min); λ=700 nm; 25 °C.

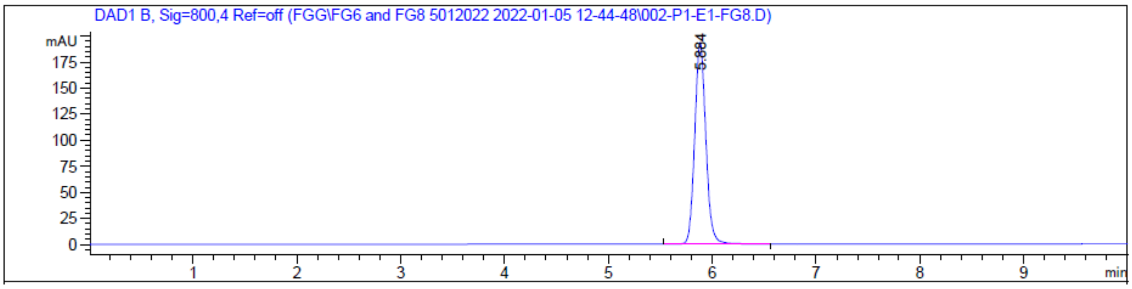

Figure S14. HPLC profile of compound **FG8**. Conditions: HPLC column: Buckyprep (4.6ID x 250 mm)), Toluene 100% as eluent (1mL/min); λ=700 nm; 25 °C.

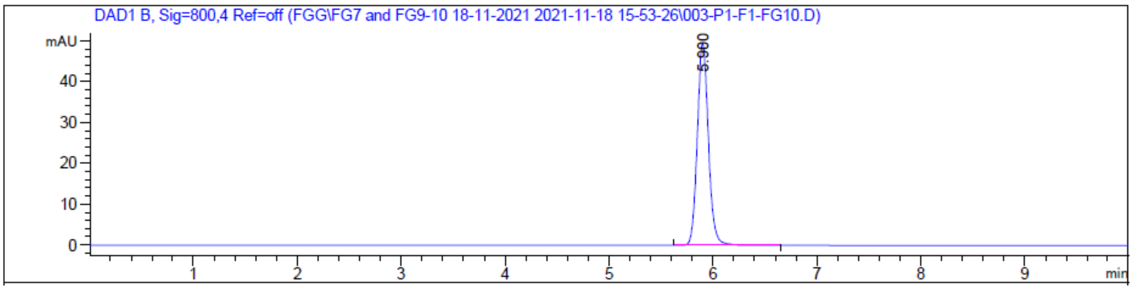

Figure S15. HPLC profile of compound **FG10**. Conditions: HPLC column: Buckyprep (4.6ID x 250 mm)), Toluene 100% as eluent (1mL/min); λ=800 nm; 25 °C.

3. Thermogravimetric Analysis (TGA) of FG6, FG8 and FG10.

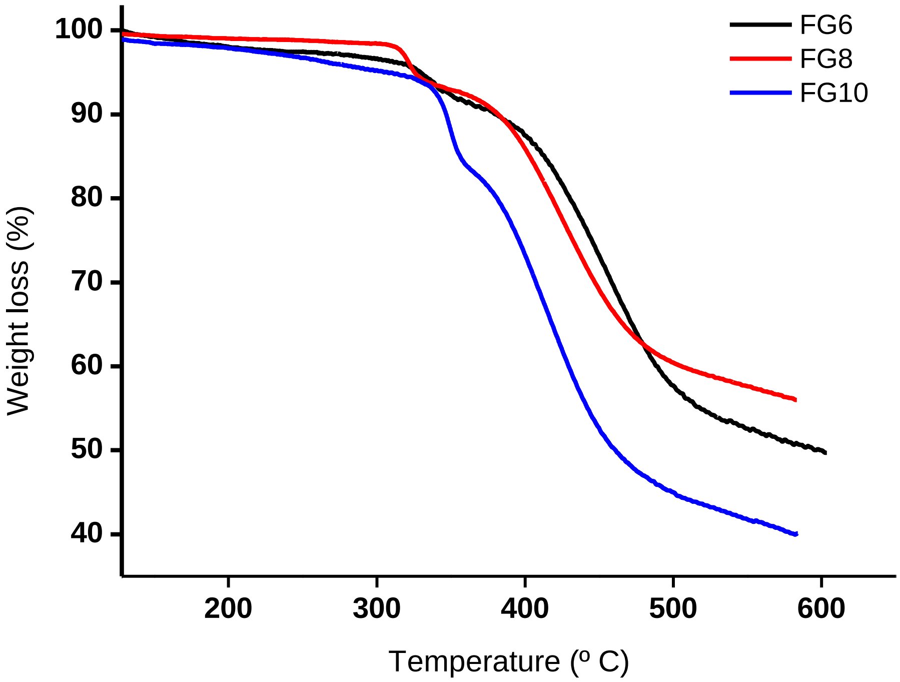

Figure S16. TGA curves of small molecule **FG6**, **FG8** and **FG10** at scan rate of 10 °C min^{-1}

Table S1. Decomposition temperatures (Td) and melting point (m.p) of **FG6**, **FG8** and **FG10**

Compound	T_d (°C)	m.p (°C)
**FG6**	329	>300
**FG8**	325	>300
**FG10**	307	278-279

4. Theoretical calculations.

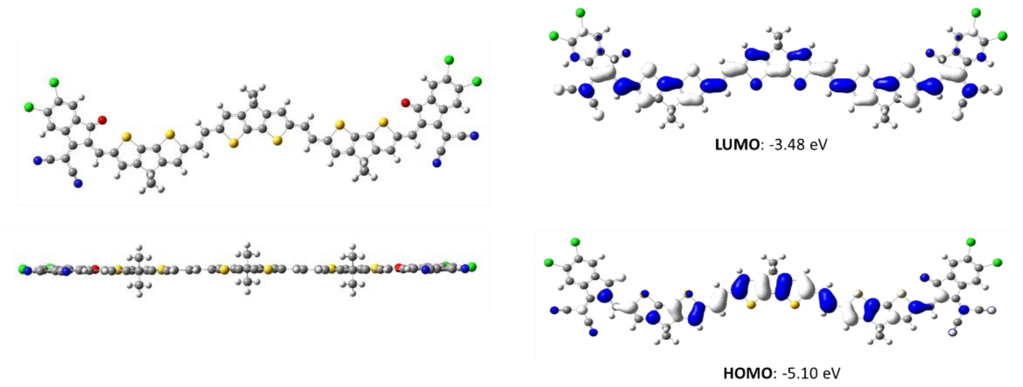

Figure S17. Optimized geometry for small molecule **FG6** (Gaussian 09W, DFT-B3LYP 6-31G)

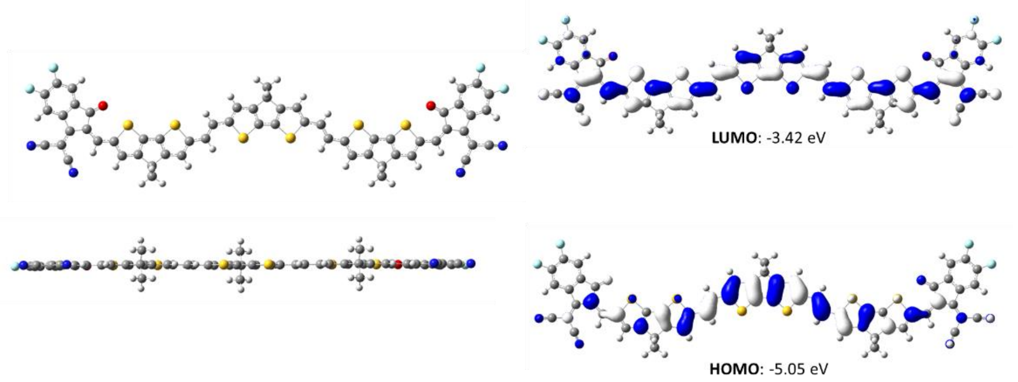

Figure S18. Optimized geometry for small molecule **FG8** (Gaussian 09W, DFT-B3LYP 6-31G)

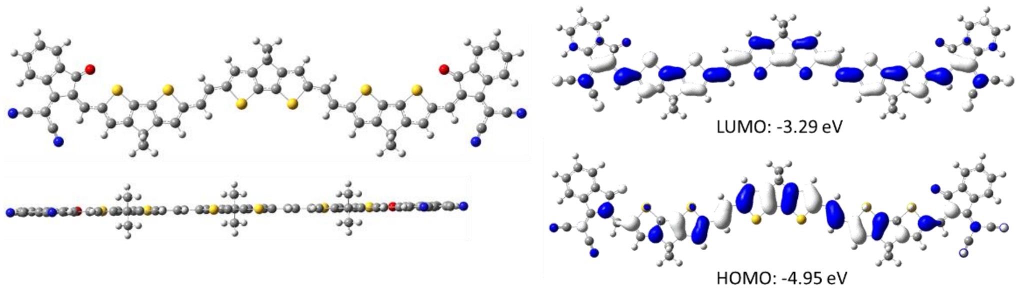

Figure S19. Optimized geometry for small molecule **FG10** (Gaussian 09W, DFT-B3LYP 6-31G)

5. Absorption spectra in solution.

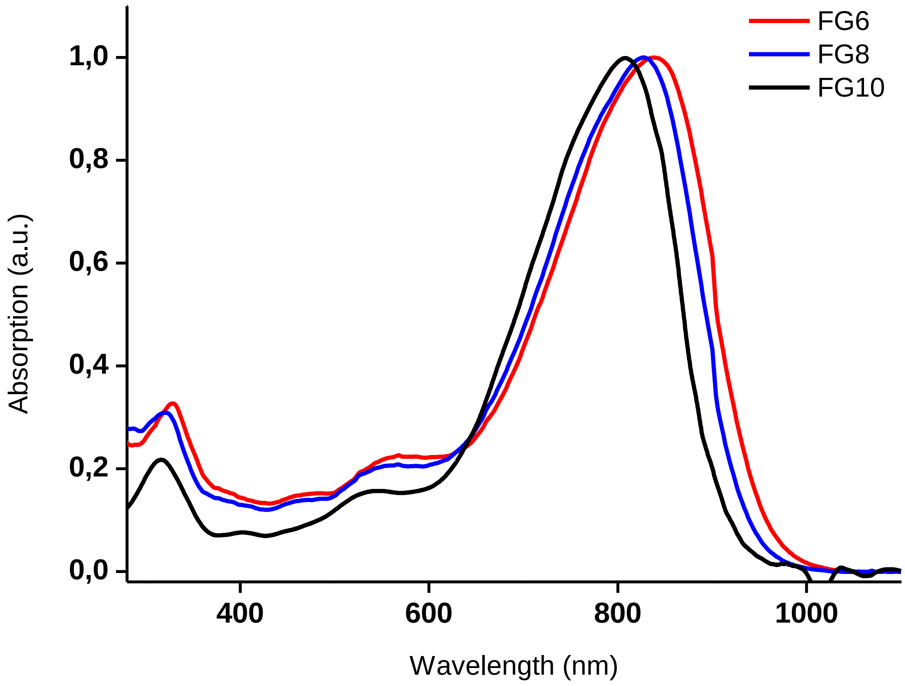

Figure S20. Normalized absorption spectra of **FG6**, **FG8** and **FG10** in chloroform solution at 1.8x10^{-6}M

6. Electrochemical Studies.

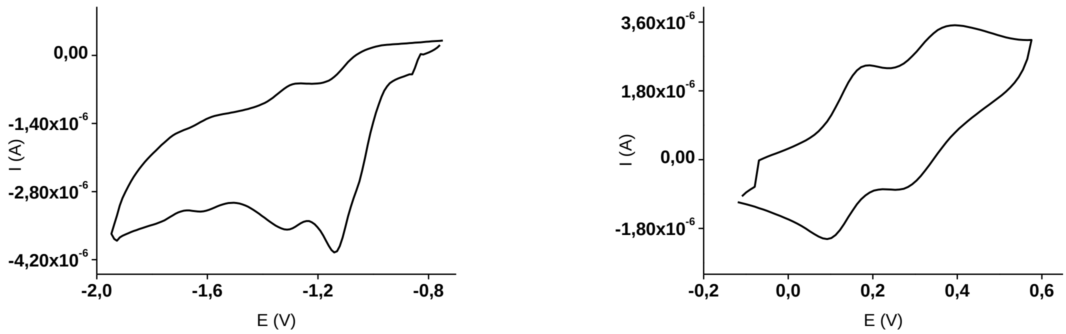

Figure S21. Cyclic Voltammetry for small molecule **FG6**: Reduction (left) and Oxidation (Right)

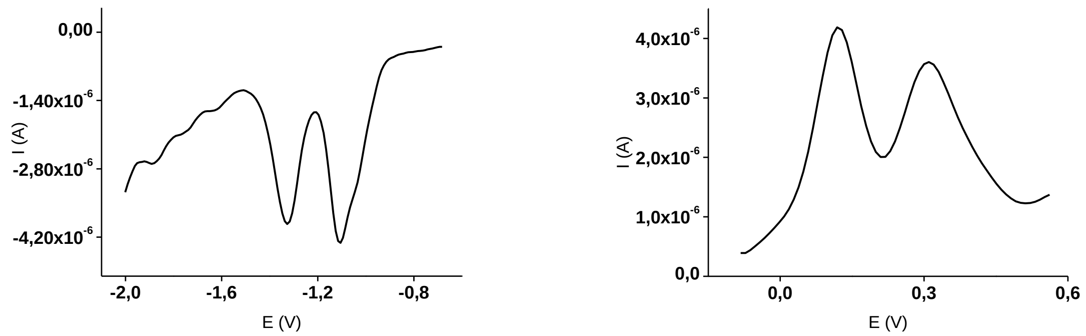

Figure S22. Oyster-Young Square Wave Voltammetry for small molecule **FG6**: Reduction (left) and Oxidation (Right)

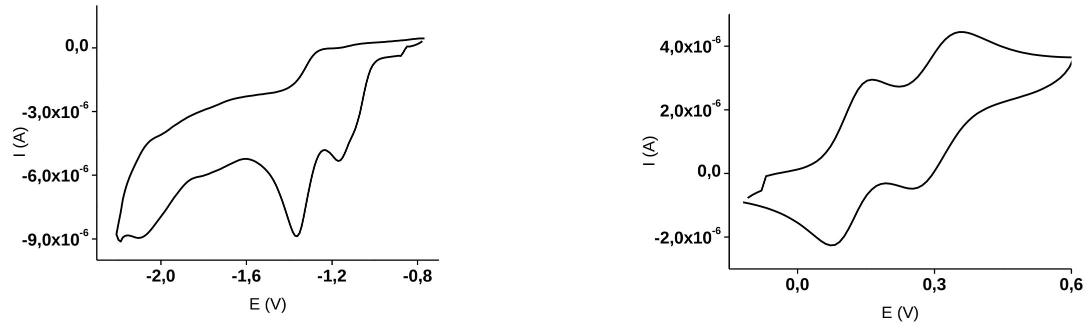

Figure S23. Cyclic Voltammetry for small molecule **FG8**: Reduction (left) and Oxidation (Right)

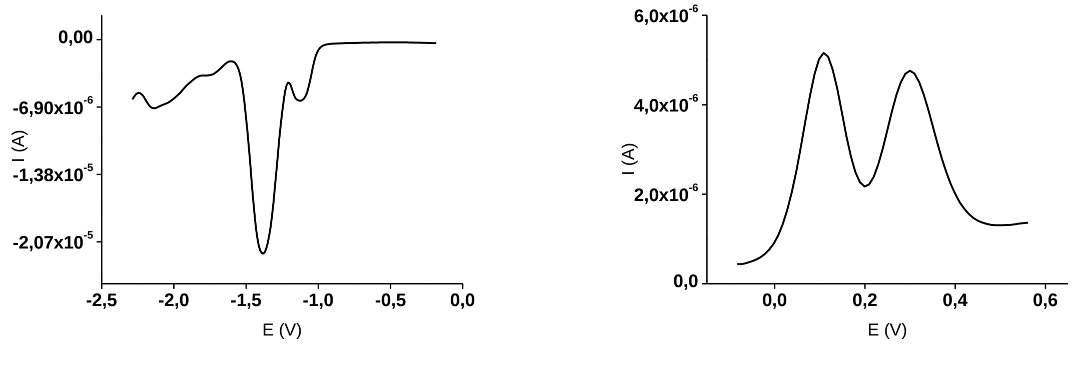

Figure S24. Oyster-Young Square Wave Voltammetry for small molecule **FG8**: Reduction (left) and Oxidation (Right)

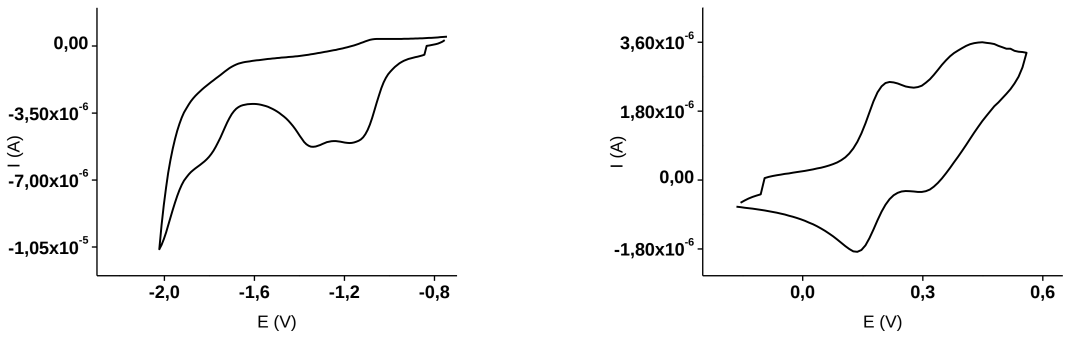

Figure S25. Oyster-Young Square Wave Voltammetry for small molecule **FG10**: Reduction (left) and Oxidation (Right)

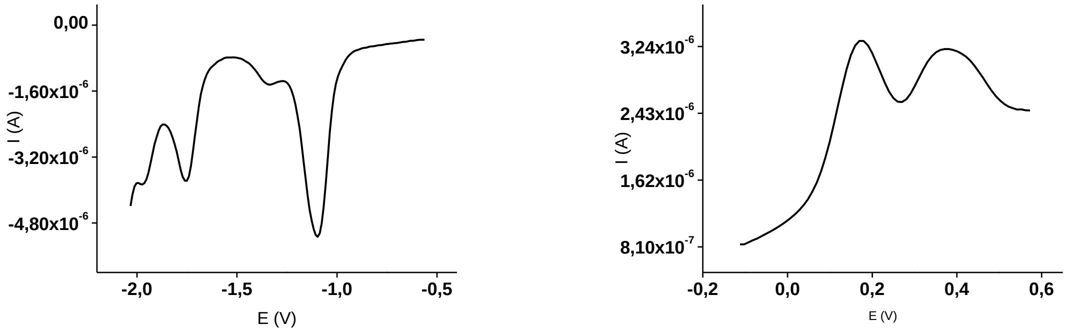

Figure S26. Oyster-Young Square Wave Voltammetry for small molecule **FG10**: Reduction (left) and Oxidation (Right)

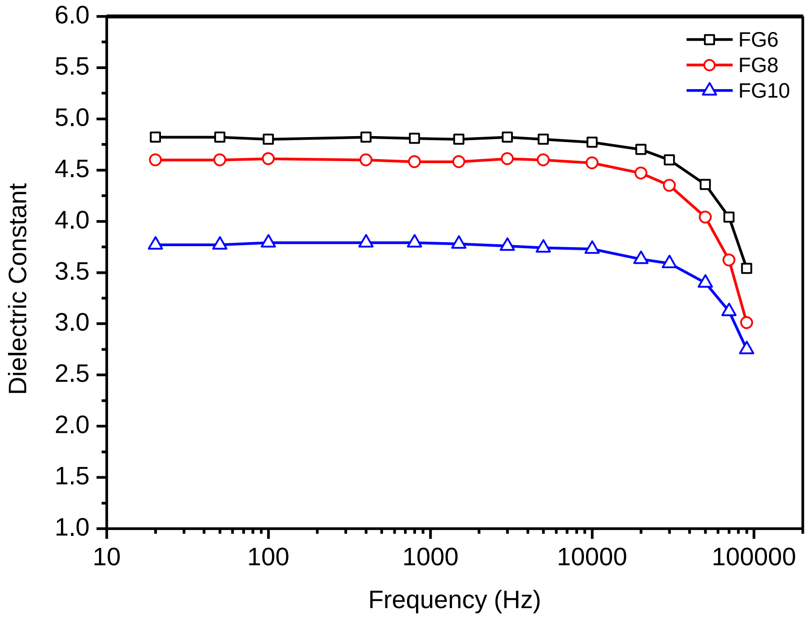

Figure S27. Variation of dielectric constant with frequency for pristine acceptor films.

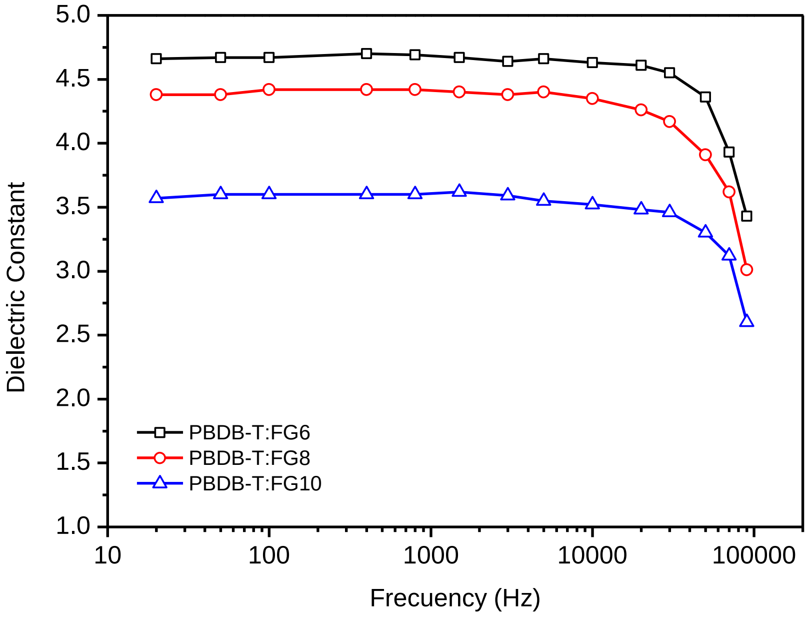

Figure S28. Variation of dielectric constant with frequency for blended films.

7. XRD data

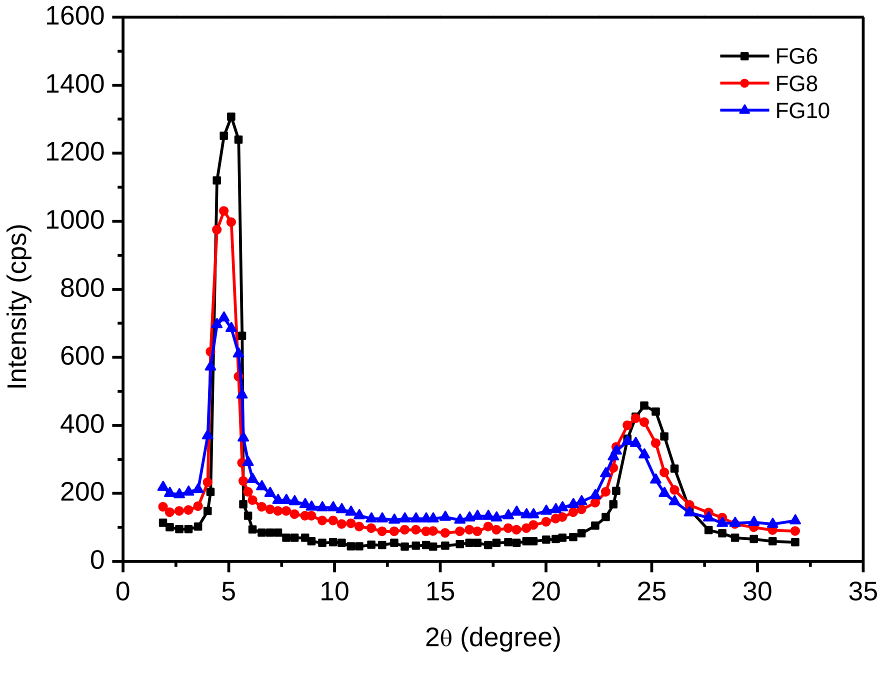

Figure S29. XRD pattern of pristine **FG6**, **FG8** and **FG10.**

8. Photovoltaic data

Table S2. Photovoltaic parameters for the PBDB-T:**FG6** OSCs with different weight ratios between PBDB-T and **FG6** processed with toluene.

PBDB-T:**FG6**	J_{SC} (mA/cm^2)	V_{OC} (V)	FF	PCE (%)
1:0.4	15.94	0.91	0.54	7.83
1:0.8	17.68	0.90	0.58	9.23
1.1.2	18.62	0.92	0.62	10.62
1:1.4	18.24	0.91	0.60	10.01

Table S3. Photovoltaic parameters for the PBDB-T:**FG8** OSCs with different weight ratios between PBDB-T and **FG8** processed with toluene.

PBDB-T:**FG8**	J_{SC} (mA/cm^2)	V_{OC} (V)	FF	PCE (%)
1:0.4	13.32	0.91	0.51	6.18
1:0.8	15.65	0.90	0.56	7.89
1.1.2	16.95	0.90	0.59	9.00
1:1.4	16.36	0.91	0.57	8.49

Table S4. Photovoltaic parameters for the PBDB-T:**FG10** OSCs with different weight ratios between PBDB-T and **FG10** processed with toluene.

PBDB-T:**FG10**	J_{SC} (mA/cm^2)	V_{OC} (V)	FF	PCE (%)
1:0.4	9.34	0.93	0.47	6.18
1:0.8	11.28	0.93	0.51	7.89
1.1.2	12.84	0.94	0.53	9.00
1:1.4	12.38	0.93	0.51	8.49
